# Impact of Spatial Soil and Climate Input Data Aggregation on Regional Yield Simulations

**DOI:** 10.1371/journal.pone.0151782

**Published:** 2016-04-07

**Authors:** Holger Hoffmann, Gang Zhao, Senthold Asseng, Marco Bindi, Christian Biernath, Julie Constantin, Elsa Coucheney, Rene Dechow, Luca Doro, Henrik Eckersten, Thomas Gaiser, Balázs Grosz, Florian Heinlein, Belay T. Kassie, Kurt-Christian Kersebaum, Christian Klein, Matthias Kuhnert, Elisabet Lewan, Marco Moriondo, Claas Nendel, Eckart Priesack, Helene Raynal, Pier P. Roggero, Reimund P. Rötter, Stefan Siebert, Xenia Specka, Fulu Tao, Edmar Teixeira, Giacomo Trombi, Daniel Wallach, Lutz Weihermüller, Jagadeesh Yeluripati, Frank Ewert

**Affiliations:** 1 Crop Science Group, INRES, University of Bonn, Bonn, Germany; 2 Agricultural & Biological Engineering Department, University of Florida, Gainesville, Florida, United States of America; 3 Department of Agri-food Production and Environmental Sciences, University of Florence, Florence, Italy; 4 Institute of Biochemical Plant Pathology, German Research Center for Environmental Health, Helmholtz Zentrum München, Neuherberg, Germany; 5 INRA, Auzeville, France; 6 Department of Soil and Environment, Swedish University of Agricultural Sciences, Uppsala, Sweden; 7 Thünen-Institute of Climate-Smart-Agriculture, Braunschweig, Germany; 8 Desertification Research Group, Universitá degli Studi di Sassari, Sassari, Italy; 9 Department of Crop Production Ecology, Swedish University of Agricultural Sciences, Uppsala, Sweden; 10 Institute of Landscape Systems Analysis, Leibniz Centre for Agricultural Landscape Research, Müncheberg, Germany; 11 Institute of Biological and Environmental Sciences, School of Biological Sciences, University of Aberdeen, Aberdeen, Scotland, United Kingdom; 12 CNR-Ibimet, Florence, Italy; 13 Environmental Impacts Group, Natural Resources Institute Finland (Luke), Vantaa, Finland; 14 Systems Modelling Team (Sustainable Production Group), The New Zealand Institute for Plant and Food Research Limited, Canterbury Agriculture & Science Centre, Lincoln, New Zealand; 15 Agrosphere Institute (IBG-3), Forschungszentrum Jülich GmbH, Jülich, Germany; 16 The James Hutton Institute, Craigiebuckler, Aberdeen, Scotland, United Kingdom; College of Agricultural Sciences, UNITED STATES

## Abstract

We show the error in water-limited yields simulated by crop models which is associated with spatially aggregated soil and climate input data. Crop simulations at large scales (regional, national, continental) frequently use input data of low resolution. Therefore, climate and soil data are often generated via averaging and sampling by area majority. This may bias simulated yields at large scales, varying largely across models. Thus, we evaluated the error associated with spatially aggregated soil and climate data for 14 crop models. Yields of winter wheat and silage maize were simulated under water-limited production conditions. We calculated this error from crop yields simulated at spatial resolutions from 1 to 100 km for the state of North Rhine-Westphalia, Germany. Most models showed yields biased by <15% when aggregating only soil data. The relative mean absolute error (rMAE) of most models using aggregated soil data was in the range or larger than the inter-annual or inter-model variability in yields. This error increased further when both climate and soil data were aggregated. Distinct error patterns indicate that the rMAE may be estimated from few soil variables. Illustrating the range of these aggregation effects across models, this study is a first step towards an ex-ante assessment of aggregation errors in large-scale simulations.

## Introduction

Process-based crop models are increasingly used for large-scale simulations at regional [1] and global scale [2]. However, these models have typically been developed at the field scale, where model driving variables are easily obtained [3–4]. Using crop models at scales other than those they were developed for may lead to biased simulations for two reasons. Firstly, biophysical processes or phenomena often depend on the scale [5–6]. Models built to simulate these processes at a given scale may therefore not be valid when changing the scale [7]. Secondly, changing the spatial resolution by aggregating or disaggregating data bears the risk of biased simulations due to modified data [8–10]. This is the so-called nonlinear aggregation error [8] or aggregation effect [11–12]. In practice, simulation studies often rely on data of different types and resolutions, disaggregated or aggregated by different methods to one resolution [13–14]. The error associated with this practice is, however, rarely considered when assessing model validity [15–16].

Large-scale yield simulations with process-based crop models rely heavily on climate and soil data inputs. Spatial resolution and combination of these inputs are known to have a large impact on the model output [17]. However, these inputs are often only available at low spatial resolution or are aggregated by spatially averaging climate [18–19] and selecting the soil type with the largest spatial coverage within a larger area [20–21]. Despite its relevance, few studies have characterized the implications of this practice (Table 1). Most of these studies have focused on climate data aggregation. For crop yield, they report an aggregation error of up to 4.7% root mean square deviation (RMSD). However, [11–12] showed that this error differs across models. Additionally, the differences mainly depend on the analyzed spatial and temporal resolution and aggregation method. While this hampers a fair comparison across studies, the aggregation error may further differ between response variables of the same simulation study. Errors considerably larger than the reported regional biases (Table 1) may therefore occur under different conditions. For instance, [10] reported errors in annual net primary production (NPP) of up to 18%, using an environmental model. As a consequence, data aggregation poses an additional complex problem when estimating model outcome uncertainty.

**Table 1:** Publications focusing on the effect of spatial input data aggregation on crop and environmental model output variables. Input data variables: climate (c), soil (s), phenology (p), management (m), topography (t), land-use (lu), vegetation (v). Aggregation methods: spatial average (av), area majority (m), direct use of maps at given resolution (map), other/various (v). Crops: winter wheat (ww), silage maize (sm), grain maize (gm), spring barley (sb). Model type: crop (c), ecosystem (e), energy balance (r).

Region^a^	Aggregation		Resolution or spatial scale	Model,no.	Response variable	Error	Reference
	data	method					
FI	c	av	10..100 km	c, 4	sb yield	Bias ^c^^,^^d^^,^^e^ ≤1.5%	[15]
DE, FR	c, s	av, map	10..50 km	c, 1	gm, ww yield	RMSE^d^ ≤4.7%	[26]
AU	c ^g^	av	0.12..0.96 km	r, 1	ETa ^h^	Bias ^c^ ≤15%	[27]
NRW^b^	c	av	1..100 km	c, 13	sm, ww yield	Bias ^c^ ≤ 3.5%, RMSE ≤4.5%	[11]
NRW^b^	c	av	1..100 km	c, 13	sm, ww yield	Bias ^c^ ≤ 3.5%, RMSE ≤4.5%	[12]
DE	c	av	1..100 km	c, 1	ww yield	RMSE^d^ ≤5%	[28]
NRW^b^	s	map	1:5·10^4^..1:10^6^	c, 4	ww yield	Bias ^c^^,^^d^^,^^e^ ≤2.9%	[16]
DE	s	map	1:5·10^3^..1:10^5^	c, 1	nitrogen leach.	Bias ^c^^,^^d^ ≤8.6%	[29]
US	c, s	v	0.5°..2.8°	c, 1	ww, gm yield	MAE ≤0.29 (ww), ≤0.7 t ha^-1^ (gm)	[23]
DK	c, s	v	various	c, 1	ww yield	Bias ^c^^,^^d^ ≤14%	[24]
DE	c, s, p	av, m	1..100 km	c, 1	ww heat stress	Bias = 0.003 ^b^ [–]	[25]
USA	c, s, lu	av, m, v	7.5..45 km	c, 1	gm yield	Bias ^c^^,^^d^ ≤2.3%	[1]
US	c, s, t, v	av	1..110 km	e, 1	NPP ^f^	Bias = 18%	[10]
DE	c, m	av	10..100 km	c, 1	ww phenology	RMSE ≤4.4%	[30]

^a)^ country code (ISO 3166–1 alpha-2).

^b)^ North Rhine-Westphalia, DE.

^c)^ average over years, region and models.

^d)^ calculated from reference.

^e)^ based on median yield.

^f)^ net primary production.

^g)^ from remote sensing.

^h)^ actual evapotranspiration.

Unlike climate data aggregation, the error in crop yield in combination with soil input data aggregation has only been investigated by few studies. This is in contrast to the fact that soils can be a major source of spatial yield variability in temperate climate [22] and thus a source of potentially large aggregation effects. Additionally, interactions of climate and soil data aggregation have rarely been studied. Using different approaches, [23–25] analyzed the impact of aggregating both soil, climate, as well as partly their interactions for single models (EPIC, CLIMCROP, and LINTUL2). They concluded that 10 km (USA, Denmark) and 100 km (Germany) resolution are sufficient to reproduce regional yield statistics. However, the relative importance of soil and climate data for the regional yield bias differed among these studies. [23] state that soil resolution had no effect on model performance in the Great Plains (USA), while [24] found decreasing winter wheat yields and increasing irrigation demand in Denmark with decreasing soil resolution. Following input data aggregation, [25] report differences in drought stress at resolutions of up to 100 km as compared to 1 km resolution. Drought stress was overestimated in drought prone regions and underestimated in more humid regions. These findings highlight the importance of interactions between model and input data on aggregation effects. Consequently, changing model or region may result in different conclusions. [10] estimated the contribution of different data types to the regional bias of NPP due to aggregating by spatial averaging from 1 to 110 km resolution. This bias was 32% due to topography, 32 to 50% due to climate, and 17 to 34% due to averaging spatial variations in vegetation and soil water holding capacity (SWHC). However, as a direct input to the employed environmental model, [10] averaged SWHC. In contrast, most crop models rely on layer-defined soil profiles (e.g. texture and physical properties) and spatial averaging is not a suitable method. Furthermore, [10] calculated the contribution of the different data types to the regional bias by averaging each data type to a single spatial average. Unfortunately, while this approach does not reflect the current practices in large-scale crop simulations, it also does not allow showing changes in spatial patterns due to data aggregation. A comprehensive analysis of soil and climate input data aggregation is therefore essential to establish a base for evaluating the significance of crop simulation studies which involve changes in scale. [23] pointed out, that such analysis should be extended to more than one model. Nevertheless, a systematic multi-model comparison of the effects of soil and climate data aggregation as well as the interaction between soil and climate data aggregation has not been carried out so far.

This work aims at analysing the effects of spatially aggregating crop model input data (climate and soil) on simulated yield for a range of crop models. We hypothesize that, under average conditions, the aggregation effect increases with increasing drought and decreasing plant available water capacity (awc) of a soil, as suggested by [7, 16]. Finally, we assume that cropping regions characterized by the occurrence of extreme soil (very low / high water holding capacity) and climate conditions (very dry / moist) are prone to the largest aggregation errors.

## Material and Methods

### Procedure and regional focus

**Fig 1:**
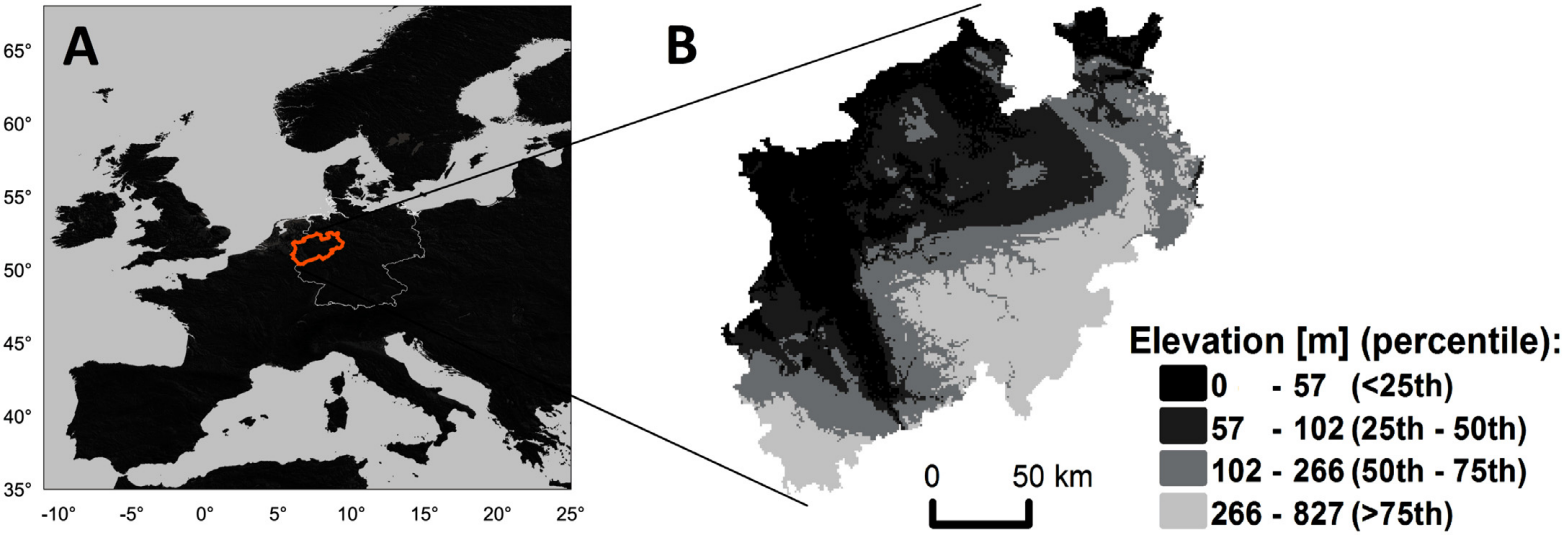
The state of North-Rhine Westphalia (NRW). (A) Location in Europe (orange line: border of NRW; white line: border of Germany). (B) Elevation above sea level.

We tested the hypothesis given above in the federal state of North-Rhine Westphalia (NRW), Germany (Fig 1A). NRW has an area of 34,098 km^2^ [31] with elevations <1000 m above sea level (Fig 1B) and a humid, temperate climate. Prevailing soil types are Cambisols, Luvisols and Stagnosols (FAO key reference soil groups: CM, LV, ST, respectively; [32]). The heterogeneous topography results in several agro-ecological zones with different soils and temperature-precipitation regimes. Spatial aggregation effects were assessed by aggregating soil and climate data to spatial resolutions varying between 1 km and 100 km. The obtained data was then used as model input for simulations of winter wheat (*Triticum aestivum* L.) and silage maize (*Zea mays* L.). Aggregation effects were estimated by relating simulated yields to climate and soil input.

### Climate and soil data processing / aggregation methods

**Fig 2:**
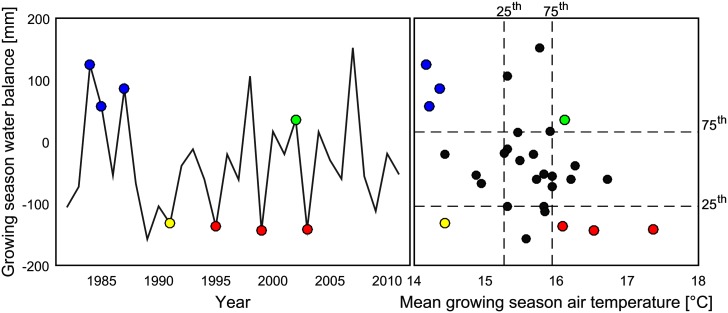
Inter-annual variability of growing season temperature (Tav) and climatic water balance of the region of North Rhine-Westphalia (regional median, calculated from 1 km resolution). Extreme years of the time series are highlighted: cold and dry (yellow), cold and wet (blue), hot and dry (red) and warm and wet (green). Extreme years are years below the 25^th^ (cold, dry) or above the 75^th^ (hot, wet) percentile of mean annual temperatures and annual precipitation, respectively.

For climate data, time series of minimum, mean and maximum air temperature (2 m above ground), precipitation, global radiation, wind speed and relative humidity were used. For this purpose, daily records from 280 weather stations and an interpolated grid of 1 km resolution of monthly records were obtained from the German Meteorological Service for the period 1982 to 2011. The station records of daily temporal resolution and the monthly grids of 1 km spatial resolution were combined to daily time series of 1 km spatial resolution following [28]. The climate properties of the region for the different spatial resolutions were given by [11]. In addition, Fig 2 shows the inter-annual variability of the climatic water balance (cwb) calculated as precipitation (P) minus reference evapotranspiration (ET): cwb = P—ET. ET was calculated according to [33].

For soil data, combinations of soil types at the scale of 1:50,000 [34] and physical parameters were joined to mapping units [16, 35]. Further soil parameters were obtained as follows: 1) Topsoil organic carbon and pH were taken from the database FIS StoBo [36]; 2) Organic carbon and C:N-ratio of deeper soil layer was approximated using pedotransfer functions [16, 35]; 3) Top soil layer C:N-ratio was set to 10; 4) Volumetric gravel content and gravel content corrected bulk density were approximated following [37–38]; 5) Soil surface albedo was estimated from soil organic carbon (Equation A in S1 File).

**Fig 3:**
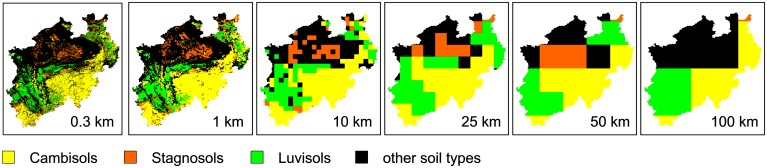
Spatial distribution of main soil types in North Rhine-Westphalia as influenced by aggregation. Resolutions of 1 to 100 km were aggregated from the source data of 0.3 km spatial resolution.

Climate and soil data were spatially aggregated in order to obtain grids of 1, 10, 25, 50 and 100 km resolution. For this purpose climate data of spatial resolution of 1 km were spatially averaged. Soil data was aggregated by selecting the dominant soil at approximately 300 m resolution raster within each grid cell of coarser resolution. A dominant soil was chosen by selecting the mapping unit of the largest area coverage (area majority). The main soil types and properties across resolutions are shown in Fig 3 and Table 2. A complete list of aggregated model input data is given by Table A in S1 Table.

**Table 2:** Main soil types in North Rhine-Westphalia as influenced by aggregation.

Resolution	No. of soil types	Cambisols	Stagnosols	Luvisols	Cambisols, Stagnosols and Luvisols
[km]	[–]	Fraction of total area [%]			
0.3	92	29.7	9.8	12.8	52.2
1	69	33.0	10.4	15.4	58.8
10	28	36.9	10.0	21.3	68.2
25	14	37.2	10.9	32.3	80.4
50	7	36.0	15.2	25.5	76.7
100	5	32.6	0.5	24.4	57.6

In addition to the described grids with a combination of soil and climate input at the respective resolution, simulations were carried out using the averaged climate time series of the entire region (mean regional climate) as well as using only one soil type representative of a typical crop land of the region (one representative soil).

All described crop model input data is available under the CREATIVE COMMONS ATTRIBUTION 4.0 INTERNATIONAL LICENSE [39].

### Crop simulations

Ensembles of models were used to simulate winter wheat (11 models) and silage maize (9 models) crop growth. These models are currently used in addressing different research questions at various scales and are described in Table 3. The models simulated the phenology, growth and yield of the crops for the period 1982 to 2011. Here, yield of winter wheat refers to grain yield whereas silage maize yield is aboveground biomass. Furthermore, both crops were evaluated for water-limited production situations, limited by crop characteristics, atmospheric CO_2_, temperature, radiation, precipitation and other soil water balance components [40–41]. A constant management except of harvest dates was used for all grid cells (Table B in S1 Table). Models were calibrated at 1 km resolution, using one typical sowing and one typical harvest date per crop in addition to the whole region area weighted average of observed yields. The latter was derived from 1999 to 2011 and from 2000 to 2008 county statistics for winter wheat and silage maize, respectively [42].

**Table 3:** Models.

No.	Model	References
1	AgroC	[43]
2	APSIM-NWHEAT ^a^	[44–46]
3	APSIM	[46–47]
4	Century	[48–49]
5	COUP ^a^	[50–52]
6	CROPSYST ^b^	[53]
7	DailyDayCent	[54–57]
8	ExpertN-SPASS ^a^^,^^b^	[58–59]
9	EPIC v. 0810	[60]
10	HERMES	[61–62]
11	MCWLA ^a^^,^^b^	[63–64]
12	MONICA	[65–66]
13	SIMPLACE<LINTUL5;SLIM>	[3, 67]
14	STICS	[68–70]

^a^ only simulated wheat.

^b^ only simulated yield at 1 km resolution.

Simulations were carried out for each grid cell for each of the following five combinations of soil and climate data grids: I) soil resolutions 1 to 100 km x mean climate; II) one select soil x climate resolutions 1 to 100 km; III) soil resolutions 1 to 100 km x climate at 1 km resolution; IV) soil at 1 km resolution x climate resolutions 1 to 100 km; V) soil x climate at same resolutions 1 x 1, 10 x 10, 25 x 25, 50 x 50 and 100 x 100 [km x km].

### Analysis

Simulated yields at resolutions >1 km were spatially disaggregated to 1 km resolution prior to further analysis. Subsequently, we calculated the agreement of the models with respect to the average yields of each cell, using an agreement indicator (Equations E and F in S1 File). Model agreement in time (years) and space (grid cells) was portrayed via a Taylor diagram [71]. Probability density functions (pdf) for yield were obtained by kernel density estimation using a Gaussian kernel (see [72] for equations). The regional bias (${\bar{Y}}_{\Delta }$) and relative mean absolute error (rMAE) of yields at coarser resolutions to yields at 1 km resolution were computed according to [11], Equation D in S1 File and Equation 4 in [12], respectively. These average statistics were based on single cell—single year differences of disaggregated coarser resolutions to a 1 km resolution. In order to obtain better insights on the direction and distribution of errors we additionally analysed these single cell—single year differences as follows:

$$\Delta_{j}=\;x_{j}^{\prime }-x_{j}$$ (1)

$$rAE_{j}=\left|\Delta_{j}\right|\cdot {\bar{x}}^{-1}$$ (2)

where Δ_*j*_ is the difference of an aggregated variable of interest disaggregated to 1 km resolution (*x*′) to the variable of interest at 1 km resolution (*x*) at a grid cell (*j)*. *rAE*_*j*_ is the relative absolute error, relative to the regional average of the variable of interest at 1 km resolution ($\bar{x}$).

In order to test how soil properties and climate regimes interact with aggregation effects, we first analysed the soil and climate input variables which explained the yield variance best over the region. Subsequently, further analysis was subdivided based on these variables. Best explaining variables were analysed by regression of average yields (1982 to 2011, median of models) of the 34168 grid cells at 1 km resolution to all possible combinations of four soil and climate input variables via PLS-regression [73]. Based on these findings (see result section), further analysis was refined for plant available water capacity awc, mean growing season temperature Tav and growing season climatic water balance cwb (Fig 2). Therefore, yields and aggregation effects were analysed separately for low and high values of the cwb and awc or Tav. High and low values were extracted by selecting values below the 25^th^ or above the 75^th^ percentile. The cwb was computed according to [33] from 1 km resolution climate data and validated by visual comparison with published maps [74] and comparison with regional mean time series of annual values (R^2^ > 0.99, Bias = −8.4 mm) with [75].

### Ethics Statement

The study did not require specific permissions. All research was carried out with publicly available data. We confirm that the field studies did not involve endangered or protected species.

## Results

### Soil and climate input data

Spatial aggregation decreased the spatial variance across climate time series (Table C in S1 Table). Averaging out climate extremes narrowed regional extremes, e.g. decreasing regional maximum annual precipitation or increasing minimum air temperature. In contrast, soil aggregation by means of majority increased the spatial variance of soil variables across the grid cells of the region (Table C in S1 Table). Despite a constantly decreasing number of soil types with aggregation (Table 2), soil variables regional minimum and maximum changed abruptly with changing spatial composition of soil types. For both aggregation methods, area mean and median were less affected (Table C in S1 Table).

### Simulated crop yields

Simulated crop yields varied across crops, years, and climate and soil conditions and in the resulting spatial pattern, as well as across models. Following we show this variability for each of these dimensions at 1 km resolution.

#### Crop

Regional crop yields (regional median, average over years) from 1 km resolution of both climate and soil data were 7.2 and 15.2 t ha^-1^ for winter wheat and silage maize, respectively (Table 4). Instead, using either the regional climate data averages or the single select soil as model input led to yields, up to 0.9 and 0.8 t ha^-1^ larger in the regional median for wheat and maize, respectively.

**Table 4:** Simulated water-limited yield at 1 km resolution over the period 1983 to 2011 and corresponding bias (${\bar{Y}}_{\Delta }$) of coarser resolutions to the yield at 1 km resolution. Values were calculated for each model first. Thereafter minimum (min), median and maximum (max) values across models were calculated. ww: Winter wheat; sm: Silage maize.

			Yield [t ha^-1^]			${\bar{Y}}_{\Delta }$ [%]											
	Model input*		1 km			10 km			25 km			50 km			100 km		
Crop	Agr.	Constant	min	median	max	min	median	max	min	median	max	min	median	max	min	median	max
ww	s	c1	6.1	7.2	7.7	-0.6	0.3	1.8	-2.9	-1.2	0.8	0.1	0.8	6.3	-8.8	-0.1	17.3
ww	c	s1	6.1	7.2	7.7	-0.8	-0.2	1.6	-0.1	0.3	2	0.0	0.9	2.3	0.3	1.7	3.8
ww	s, c	-	6.1	7.2	7.7	-1.2	0.1	1.9	-2.6	-0.6	1.9	0.9	1.9	7.6	-7.9	1.3	17.8
ww	s	cNRW	6.1	7.6	8.4	-0.7	0.0	2.8	-3.3	-1.1	0.7	-1.8	0.9	3.7	-5.0	-0.4	16.9
ww	c	sNRW	7.0	8.1	9.3	-0.5	0.0	0.6	0.1	0.7	1.2	0.5	1.1	2.1	0.7	1.1	3.3
sm	s	c1	10.4	15.2	17.7	-0.8	0.2	1.6	-2.3	-0.7	1.3	0.1	1.2	5.2	-7.4	0.9	42.7
sm	c	s1	10.4	15.2	17.7	-1.0	-0.1	3.3	-0.7	0.7	3.8	-0.2	1.4	4.5	-3.1	2.3	6.1
sm	s, c	-	10.4	15.2	17.7	-1.3	0.4	3.5	-2.1	0.7	3.1	0.9	3.1	7.9	-5.7	2.1	18.5
sm	s	cNRW	11.3	15.3	19.8	-0.8	-0.1	2.1	-2.5	-1.0	0.6	-0.6	0.7	4.3	-2.6	0.9	16.7
sm	c	sNRW	13.1	16.0	20.5	-1.0	0.1	0.8	-0.6	0.3	1.2	-0.3	0.6	1.7	-1.5	0.9	2.6

*) Abbreviations refer to soil (s, s1, sNRW) or climate (c, c1, cNRW) model input. Agr: Aggregated to 10, 25, 50 or 100 km resolution. Constant: Not aggregated model input kept constant at 1 km resolution (c1, s1) or using an average climate time series (cNRW) or one soil for the region (sNRW). Example: Values for aggregated soil (s) and constant climate at 1 km resolution (c1) are min/median/max yields from simulations with soil resolutions of 10 to 100 km in combination with climate data of 1 km resolution.

#### Temporal variability

The regional yields showed an inter-annual variability (mean of year-to-year standard deviation from each grid cell and model) of 1.1 t ha^-1^ (15.3%) and 2.4 t ha^-1^ (15.8%) for winter wheat and silage maize, respectively. Yields from simulations with spatially averaged climate (1.0 and 2.2 t ha^-1^) or with one select soil (0.8 and 1.9 t ha^-1^) showed an inter-annual yield variability of a similar range. In the case of winter wheat, however, regional yields ranged mainly from 7 to 8 t ha^-1^ with considerably lower yields in 2010 and 2011 (Figure B in S1 Fig).

#### Spatial variability

We found, that the spatial yield variability could be explained by 68% (winter wheat) and 81% (silage maize) based on four variables (Figure A in S1 Fig): growing season precipitation, plant available water capacity of the soil profile (awc, [mm]), soil profile depth and topsoil awc in the case of wheat and growing season mean daily temperature, awc, soil profile depth and topsoil awc in the case of silage maize. The single variable explaining most of the spatial variability was awc, (38% wheat, 58% maize), followed by soil profile depth (33% wheat, 36% maize). Climate variables showed low explanation power for spatial variability for wheat (<10%), whereas spatial variability of average silage maize yields were explained 31, 35 and 36% by average daily minimum, mean and maximum air temperature, respectively. As a consequence, the further analysis was refined (see methods) for awc, Tav and cwb of the growing season.

In contrast to the year-to-year fluctuation in yield, the spatial variability in yields depended more on the spatial input data resolution. For instance, the mean of cell-to-cell standard deviation from each year and model from both climate and soil input data at 1 km resolution was 1.3 and 3.1 t ha^-1^, for winter wheat and silage maize, respectively. These values decreased when using the regional climate data averages (1.0 and 2.2 t ha^-1^) or the single select soil (0.6 and 1.7 t ha^-1^) as model input. Thus, substituting high resolution input data by a single soil decreased the spatial variability more than substitution by a single climate time series.

**Fig 4:**
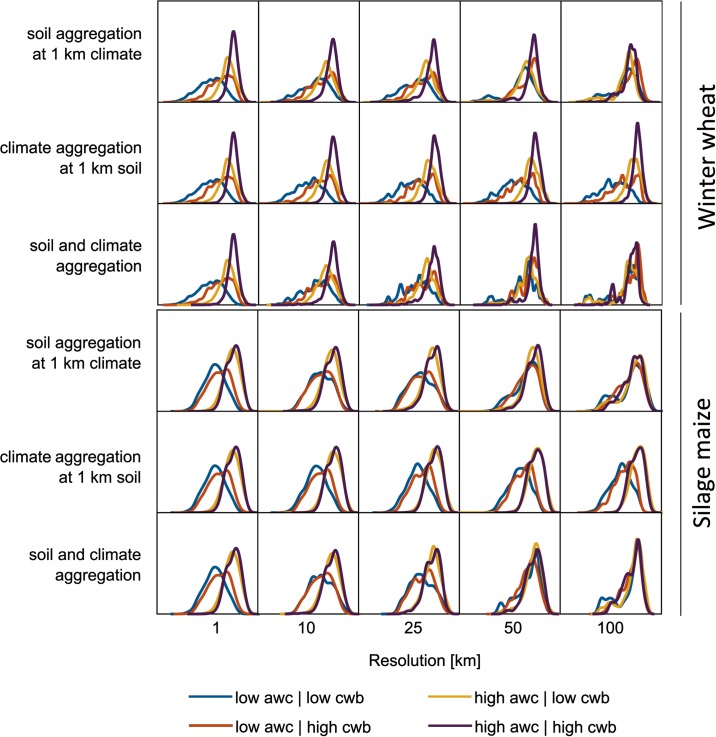
Simulated probability density function (pdf) of winter wheat and silage maize yield under water-limited conditions in North Rhine-Westphalia, Germany (1983–2011) as related to the aggregation of different data types as well as the plant available water capacity (awc) over the profile and climatic water balance (cwb) of the growing season. ‘low’ and ‘high’ refer to values below the 25^th^ or above the 75^th^ percentile. Pdf was estimated via kernel density (Gaussian kernel with bandwidth of 0.1 and 0.5 for wheat and maize, respectively). Subplots range from 2 to 10 t ha^-1^ and 1 to 21 ha^-1^ for wheat and maize, respectively.

Single year crop yields ranged from 2.5 to 9.5 t ha^-1^ and from 4 to 20 t ha^-1^ for winter wheat and silage maize respectively, when averaged across models (Figure C in S1 Fig). While silage maize yields for years with a growing season cwb above -200 mm increased with increasing awc, winter wheat yields continuously increased with both awc and cwb. In this context, winter wheat yields above 8.5 t ha^-1^ were mainly reached above 300 mm growing season cwb and on soils with awc >100 mm. Consistently, winter wheat yields below 7.5 t ha^-1^ occurred mainly with negative cwb and on soils with awc <200 mm. Silage maize yields of 16 t ha^-1^ were achieved on soils with awc >200 mm, regardless of cwb. Accordingly, yields showed characteristic probability density functions at upper and lower quartiles of awc and cwb (Fig 4).

**Fig 5:**
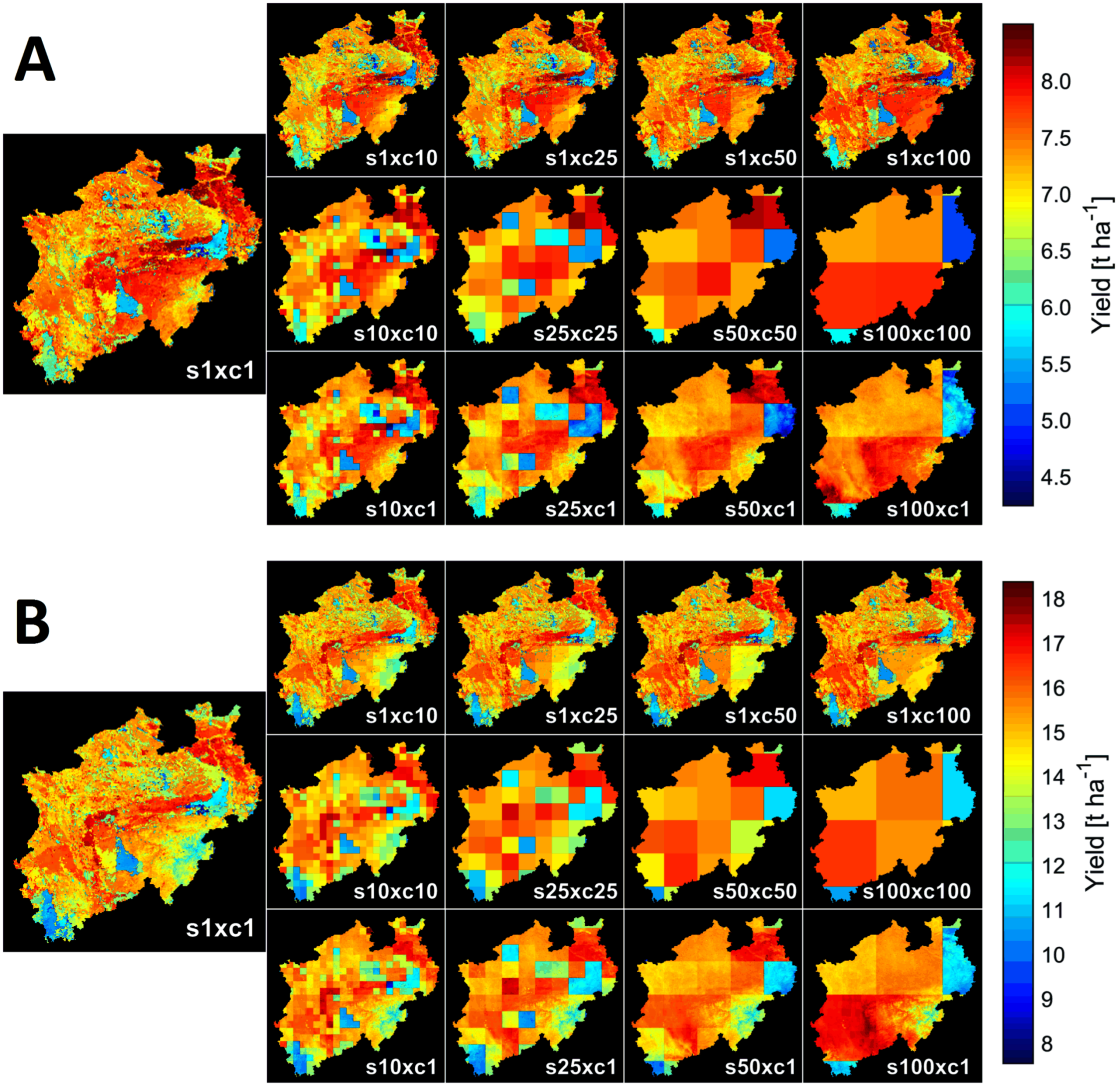
Simulated water-limited yield of North Rhine-Westphalia, Germany (1983–2011; mean of years and models). (A) Winter wheat. (B) Silage maize. Results are shown for a range of model input data combinations of soil (s) and climate (c) data of 1, 10, 25, 50 and 100 km resolution.

The interaction of soil and climate conditions was reflected in the spatial pattern of the crop yields (Fig 5). With 8.7% and 90.6% of all grid cells at 1 km resolution exhibiting soil profile depths of ≤0.6 m and ≥1.7 m, respectively, the comparatively small fraction of shallow soils (≤0.6 m) led to characteristic spatial yield patterns. For instance, areas of low yield were found in the north-east, central south, and south-west of NRW, coinciding with shallow soils. These shallow soils exhibited low awc and were mainly located at higher elevations (Fig 1). Unlike these differences in specific soil variables of a given soil type, average yields across soil types differed less. From the three main soil types of the region (Fig 3), the average yields over years were (mean ± standard deviation over the region) 7.1± 0.8, 7.5 ± 0.5, 7.2 ± 0.7 t ha^-1^ for winter wheat and 13.9 ± 1.8, 15.9 ± 1.1, 14.7 ± 1.3 t ha^-1^ for silage maize, respectively for Cambisols, Luvisols and Stagnosols. Highest average yields were found on gleyed Cambisols and Chernozem-Luvisols (>8 t ha^-1^ winter wheat; >16.9 t ha^-1^ silage maize). In contrast, yields below 6 t ha^-1^ (winter wheat) and 12 t ha^-1^ (silage maize) were found on a variety of soil types, mainly characterized by shallow profile depth and/or large gravel content.

#### Model variability

In general, models showed a larger coefficient of variation (cv) in silage maize yields (cv = 27%) than for winter wheat yields (cv = 18%). This is illustrated by the scatter of models in Taylor plots (Figure D in S1 Fig). Average yields of years and region ranged from 6.1 to 7.7 t ha^-1^ and from 10.4 to 17.7 t ha^-1^ for winter wheat and silage maize, respectively. In addition, the variability across models for single years and cells was generally comparable or larger than the inter-annual variability or variability of yields across the region. For instance, the standard deviation across crop yields for each cell and year was 1.4 and 4.1 t ha^-1^ on average for winter wheat and silage maize, respectively. However, in contrast to the spatial variability, model variability did only decrease when using one single soil as model input (1.0 and 3.4 t ha^-1^), but not when using an average climate time series as input (1.4 and 4.0 t ha^-1^). In general, models agreed better with increasing yield. This is partially reflected in the spatial pattern of model agreement (Figure E in S1 Fig). Characteristic patches of shallow soils can be distinguished as linked to low model agreement. Thus, models agreed less on winter wheat yields and silage maize biomass on shallow soils.

### Yield aggregation effects

Yields simulated with aggregated input data differed from yields simulated at 1 km resolution (Table 4). The regional yield bias (${\bar{Y}}_{\Delta }$) was up to ±3.1% of yields at 1 km resolution (median of models, Table 4), but differed considerably under specific conditions as shown in the following.

#### Crop-specific aggregation effects

Relative to the regional mean yield at 1 km resolution, maximum ${\bar{Y}}_{\Delta }$ for both crops were comparable in the median of models. In more detail, differences between the two crops were found for single resolutions and input data combinations. For instance, ${\bar{Y}}_{\Delta }$ of opposite signs were found between the crops with both, soil and climate data, aggregated to 25 km resolution. However, differences in model median biases across all resolutions were relatively small as compared to the spread across models (see min and max ${\bar{Y}}_{\Delta }$, Table 4).

#### Temporal variability

**Fig 6:**
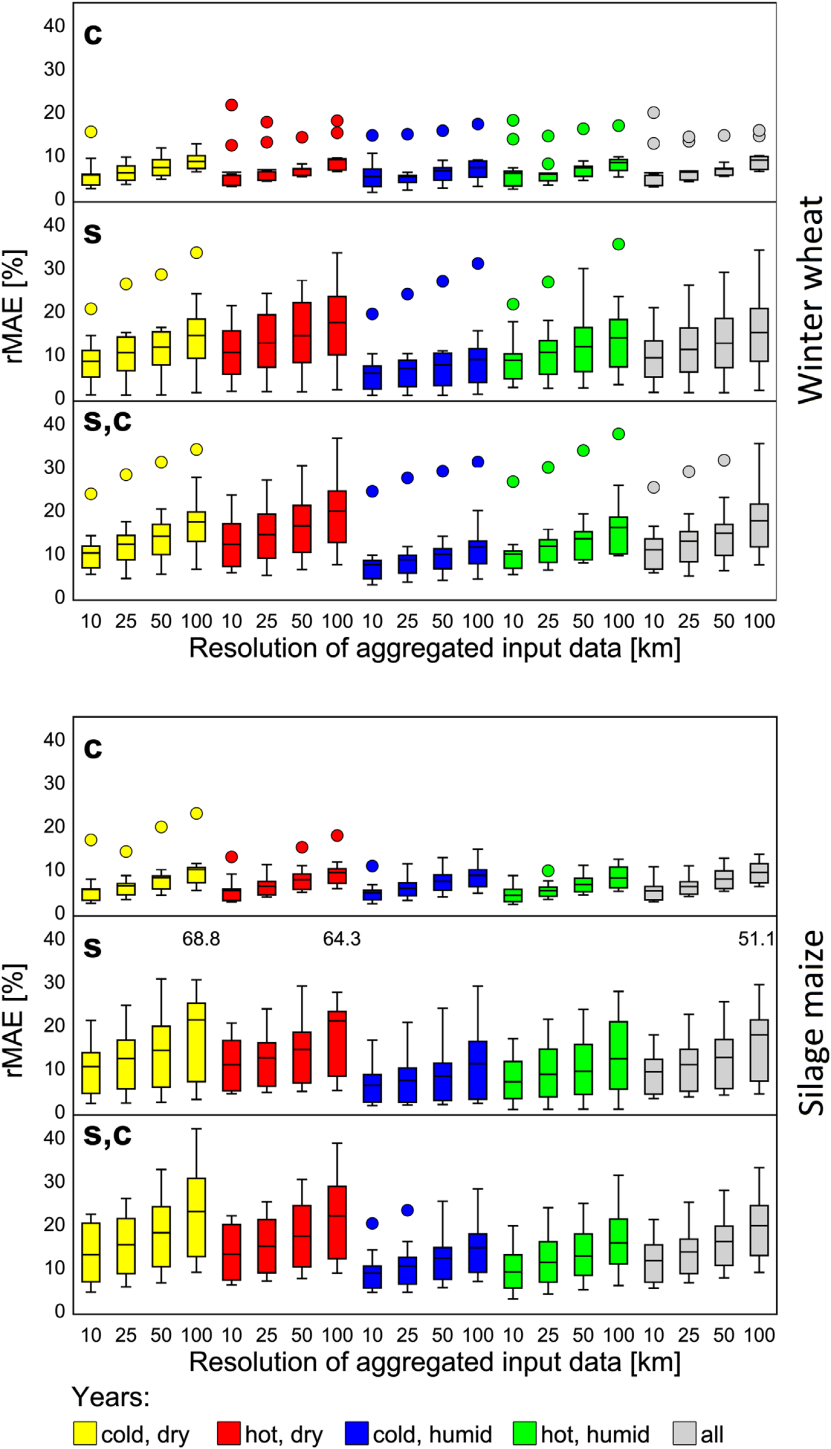
Relative mean absolute error (rMAE) of simulated winter wheat and silage maize yield under water-limited conditions for different spatial resolutions of model input data. **c**: aggregated climate x 1 km soil; **s**: aggregated soil x 1 km climate; **s,c**: aggregated soil x aggregated climate. The rMAE was calculated from data of extreme years (see Fig 2) and of all single years. Boxplots show the rMAE calculated from single model results (winter wheat: n = 11; silage maize: n = 9). The middle line indicates the mean rMAE across models. Whiskers are Tukey style and extent to 1.5 times the interquartile range (see [76]). Extreme outliers are indicated by written numbers.

Regional biases varied less than 0.2 t ha^-1^ (≤1.6%; standard deviation) across years, regardless of resolution or data input combination (see also Figure B in S1 Fig). However, the inter-annual variability of single cells’ yield deviation from yields at 1 km resolution was in the range of the single cells inter-annual variability at 1 km resolution. In this case, the standard deviation across all years was 0.8 and 1.7 t ha^-1^ for winter wheat and silage maize, respectively, as compared to the standard deviation of yields of 1.1 and 2.4 t ha^-1^, respectively. However, the rMAE was increased in years of lowest growing season cwb as compared to those of highest cwb in all input data combinations with aggregated soil data (Fig 6, Figure F in S1 Fig). This larger rMAE in years with low growing season cwb was found independent of the growing season temperature.

#### Spatial variability

**Fig 7:**
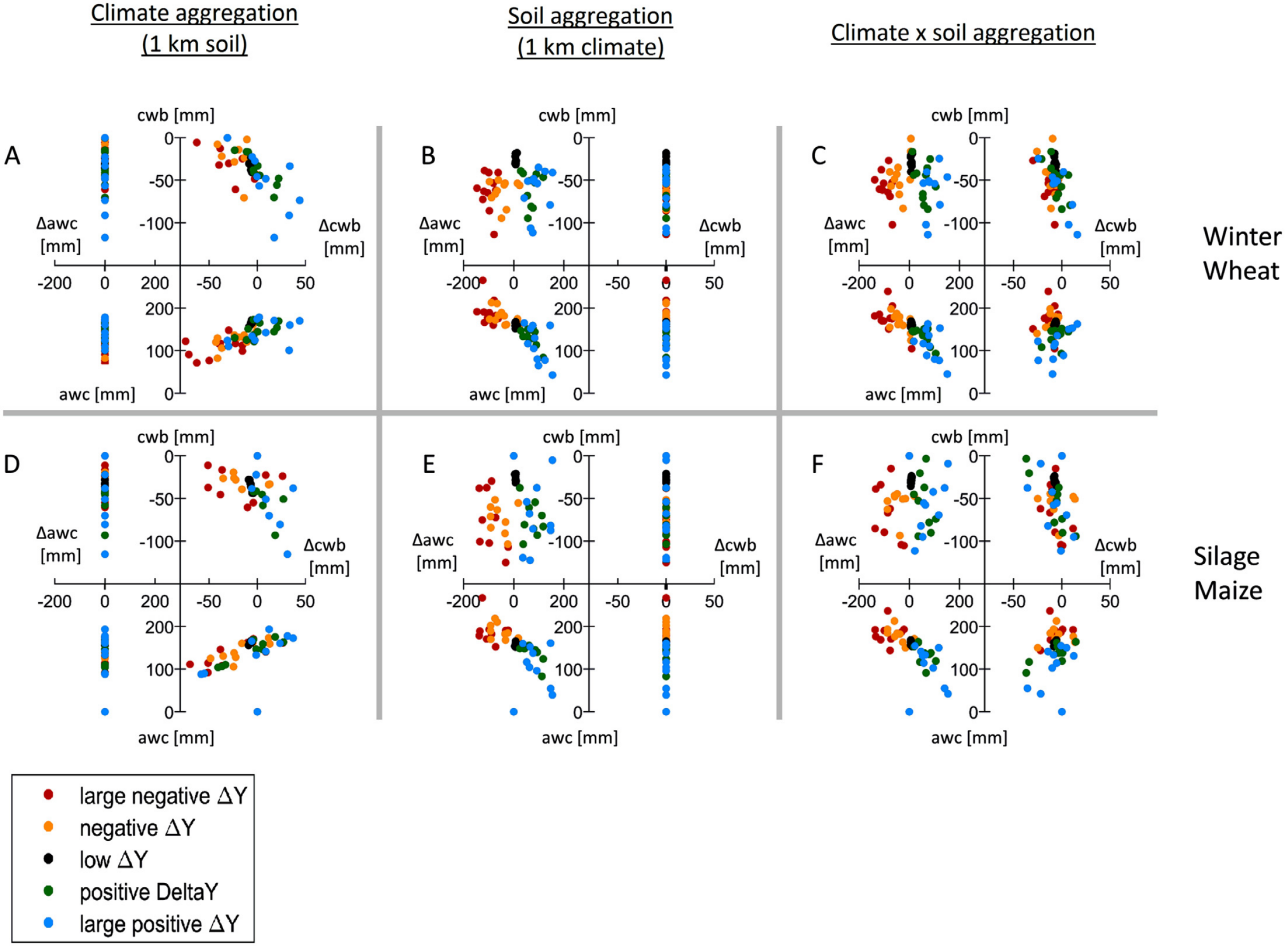
Differences in yield (ΔY) due to data aggregation as related to available water capacity (awc) and climatic water balance during the growing season (cwb) at 1 km resolution and the corresponding differences from coarser resolutions to 1 km resolution due to data aggregation (Δawc, Δcwb). Panels show results for winter wheat (A,B,C), silage maize yields (D,E,F) and three combinations of aggregated data: A,C: climate aggregated and 1 km soil resolution; B, D: soil aggregated and 1 km climate resolution; C, F: soil and climate aggregated simultaneously. Values shown are single model means for each of the five groups of aggregation effects (one scatter dot per model), grouped as follows. Large negative ΔY: ΔY<μ-2σ; negative ΔY: μ-2σ <ΔY<μ-σ; low ΔY: μ-σ <ΔY<μ+σ; positive ΔY: μ+σ <ΔY<μ+2σ; Large positive ΔY: ΔY>μ+2σ where μ: mean of ΔY and σ: standard deviation of ΔY. See Table D in S1 Table for detailed values.

**Fig 8:**
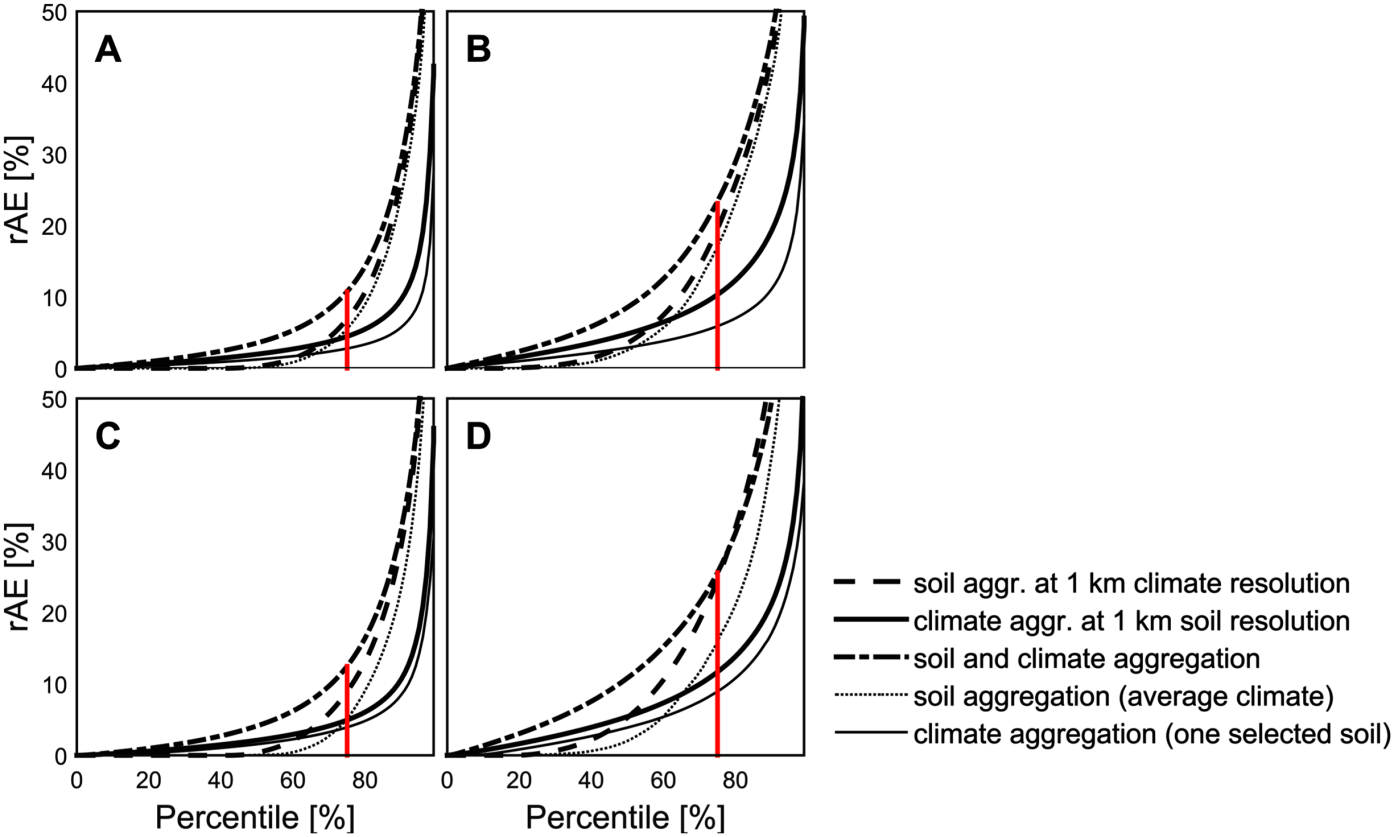
Relative absolute error (rAE) of yield under water-limited conditions from single cells as compared to 1 km resolution (n = 34168 cells x 30 years x 11 models = 11275440). (A) Winter wheat, aggregated data at 10 km resolution. (B) Winter wheat, aggregated data at 100 km resolution. (C) Silage maize, aggregated data at 10 km resolution. (D) Silage maize, aggregated data at 100 km resolution. The red line highlights the 75% percentile of combined soil and climate aggregation.

Similar to yields, also the rMAE varied considerably with type of input data. Three distinct effects were identified. Firstly, the probability density function (pdf) of yields from soils of low awc change when aggregating soil (Fig 4). At aggregations of ≥50 km, these pdf’s resemble those from high awc soils. In contrast, aggregation of climate did not show this effect. Secondly, the aggregation error increased with decreasing awc when aggregating soil data (Fig 7). Further, when aggregating soil, cwb showed no clear impact on the aggregation error. However, using aggregated climate data in combination with 1 km soil data as model input showed a contrasting pattern. In this case, aggregation effects increased with increasing cwb and with decreasing awc at 1 km resolution. These two effects partially resulted in a third effect: Aggregation effects showed distinct functions depending on the input data type and resolution (Fig 8). Aggregated soil data combined with either climate data at 1 km or averaged over the region revealed the fraction of the rAE with nearly zero error (rAE ~ 0%). For instance, the rAE at 10 km resolution was close to 0% in approximately ≥50% of all cells and years. This fraction decreased with further aggregation. In contrast, climate data aggregation caused a larger fraction of low rAE (0 ≤ rAE ≤ 10%). Consistently, soil aggregation led to a larger fraction of high rAE. Finally, the combined aggregation of soil and climate data led to distribution of rAE with both a larger fraction of low rAE as compared to soil data aggregation as well as a larger fraction of high rAE as compared to climate data aggregation.

#### Model variability

Crop models differed in their aggregation effects. While most models showed aggregation effects of similar range for a given condition, few models showed considerably larger errors under specific conditions. This is expressed in the minimum and maximum model biases (Table 4) as well as in the outliers when analysing the rMAE (Fig 6). However, these extreme results strongly depended on the specific condition (data type, resolution, year, crop, etc.).

## Discussion

### Data aggregation effects on regional soil and climate statistics

Input data were modified by aggregation, affecting the regional mean and variability of the data. The magnitude of the modification is mainly due to two factors: i) the aggregation method and ii) the spatial variability of the data at the finest resolution. Exemplarily, it was shown in the present study, how the spatial variability of climate time series decreases with spatial averaging. This is in agreement with [11] who also showed the spatial dependency of the climate data (variogram). Considering the aggregation method *averaging* and the larger spatial kriging range of most climate variables as compared to soil data, aggregated climate data was expected to exhibit continuous (“smooth”) aggregation effects with coarser resolutions. In contrast, soil data aggregation led to abrupt changes in the data statistics due to the selected method. These changes are the result of the changing mixture of soil types in space with aggregation which leads to large differences when compared to the highest resolution. Furthermore, these abrupt changes are not monotonously increasing or decreasing and the aggregated data thus depend highly on the resolution. However, effects on simulated yields may be rather small regarding regional statistics. This is in agreement with [25], who found decreasing extremes but similar 5^th^ and 95^th^ percentiles in soil water storage capacity after aggregating soil types from 1 to 100 km resolution by area majority for Germany. Thus, the prevailing spatial variability of the soil data in this study could be representative for a larger fraction of soils in Central Europe. Large-scale crop modelling studies may therefore be robust to such data artefacts on average, but prone to errors at higher resolutions, especially when using spatially aggregated soil data. For instance, the selected resolution in such studies may lead to different directions and magnitude of aggregation effects of input data in different regions. Therefore, new concepts are needed to integrate these effects into the assessment of uncertainty in large-scale studies when making statements on sub-regions or spatial patterns.

### Relationship of simulated water-limited and potential yield in aggregation effects

Soil and climate data aggregation effects on yield may be larger in extreme situations. For instance, [11] hypothesized that large positive effects on yield would be observed best in situations with high abiotic stress. In contrast, large negative effects would be observed best under near-optimum conditions. Since the modelled yields include simulations from soils and climatic periods with non-optimum conditions as well as homogeneous management, it is questionable how much abiotic stress prevailed in the present simulations. Simulated yields of 7.2 to 8.1 t ha^-1^ (winter wheat) and 15.2 to 16.0 t ha^-1^ (silage maize) in the median of models were similar to observed yields of 7.2 and 14.3 t ha^-1^ [11]. With an annual precipitation larger than 800 mm (regional median), a high degree of drought stress was not expected [11–12]. For the same investigated region, [11–12] simulated yields with a similar model ensemble for potential production situations, where crop growth was only limited by temperature and radiation input data. The yield gaps obtained between potential and water-limited conditions were 4.7 and 6.9% for winter wheat and maize respectively. In these studies, a single soil profile of high awc was applied for the entire region, equivalent to the combination of a single selected soil vs. climate data aggregation applied in the present study. Here we show regional mean yields of 0.9 t ha^-1^ (winter wheat) and 0.8 t ha^-1^ (silage maize) lower than for the mentioned single soil profile. With this single soil profile, [11–12] obtained similar yields as presented here (differences ≤0.2 t ha^-1^). Therefore, abiotic stress due to water limitation can be approximated by relating the potential yields of the mentioned studies to the water-limited yields of the present study. As a result the average yield reduction of all years due to water deficits in the present study turned out to be 15.1 and 11.5% for winter wheat and silage maize, respectively. Since water scarcity is mainly restricted to sub-regions of low awc and sporadically low cwb, larger rAE and rMAE could be expected for the present study, in particular due to differences in the soil input data.

### Effects of data aggregation on simulated yields

#### Interpretation of aggregation effects and comparison to other sources of yield variability

Using aggregated climate and/or soil model input data lead to errors in simulated water-limited yields of winter wheat and silage maize. In the present study, regional yield biases of up to ±3.1% (ensemble median) were in the range of published studies (Table 1). Furthermore, these ensemble median biases were relatively small compared to the inter-annual yield variability or variability of yields across crop models. However, two exceptions of considerably larger aggregation effects could be recognised. Firstly, few models showed considerably larger biases of up to 42.7%. In addition, model biases partially ranged from negative to positive values. For instance, model biases for silage maize yield ranged from -3.1 to +6.1% with climate input data aggregation in combination with soil data of 1 km resolution. Since the median of biases across models was +2.3% in this case, the model ensemble median seems to be a rather poor indicator of positive or negative aggregation effects. Secondly, the rMAE was considerably larger than yield biases, partially exceeding the inter-annual or inter-model variability in yields. Therefore, aggregation errors can be a dominating source of error when regarding the rMAE calculated from single years. In contrast, these errors cancel out in a long-term average. Finally, the rMAE calculated from yields averaged over the years (data not shown) compared well to the relative average absolute deviation (rAAD) reported by [12] for climate data aggregation in combination with homogeneous soil input data. This rMAE is lower than the rMAE reported here as calculated from single years. Thus, caution should be taken when interpreting results as the different error statistics report larger errors i) when calculated from single years and averaged afterwards as compared to calculations from averages of years and ii) for the rMAE than for the bias. This is illustrated by Figure G in S1 Fig, where ensemble median of regional mean yields correlated well to yields at 1 km resolution (R^2^>0.95), but single cell yields correlated poorly to yields at 1 km resolution (R^2^≤0.31) when aggregating both soil and climate input data.

#### Assessing regional soil and climate data aggregation effects

Aggregation effects have been investigated by a range of studies (Table 1). In order to relate these findings to the present study, the following steps were applied: 1) aggregation of one type of input data keeping other information constant at coarse resolution (e.g. one data input value per region); 2) aggregation of one type of input data keeping other information constant at high resolution (e.g. 1 km); 3) aggregating several types of input data simultaneously.

While step 1 aims at analysing the aggregation effect of one data type, it fails at assessing the interactions with the data type, which is not aggregated for realistic conditions. For instance, climate input data aggregation effects have been investigated by using a single soil profile across all simulations [11–12, 15, 28]. This is comparable to the single soil vs. climate aggregation of the present work. All of these studies agree well with the present work for low regional yield bias, being considerably lower as compared to inter-annual variability or model differences in yields. This suggests that climate data aggregation contributes less to the uncertainty in upscaling for yield simulations. However, these studies were carried out with temperate climates of positive annual water balances and typical agricultural soils of awc above 200 mm. Thus, these studies may not be suitable to portray the full variability or aggregation effects in other more-constraint climate environments. Using aggregated soils with average climate or single climate time series is analogue to this.

In contrast, the steps 2 and 3 given above account for the entire range of input data variability of a region. For instance, [10] aggregated different input data types of high resolution for an ecosystem model by averaging. By aggregating the different data types stepwise, they were able to quantify the contribution of each data type to the aggregation bias. But, except for single specific soil variables (e.g. awc), averaging soil information is usually not meaningful, since averaging may lead to strong inconsistencies among different variables. This is due to the nonlinear relations of the soil variables and the impact of averaging on texture classes, e.g. by averaging sand and clay fractions. And, when using different aggregation methods, effects on yield are not necessarily additive. Therefore, aggregation effects across soil and climate can only be assessed by comparing systematically outputs from simultaneously aggregated inputs with outputs from separately aggregated inputs (keeping one input data type constant) and simulating a large range of possible input data combinations (large spatial variability) for various resolutions. To do so [24] used CLIMCROP to simulate winter wheat yields with “fine resolution” soil data (Danish soil survey) as well as with one representative soil per county, keeping climate data at 1 km resolution. A maximum bias of 13.9% of simulated to observed yields was reported. Although this finding is not directly comparable to the present study due to the different resolutions, grids and underlying data used to construct the grids, it is similar to biases found here.

It is also possible to derive the optimum data resolution by knowing the regional yield bias. Therefore, [24] concluded that a minimum soil resolution of 10 km is needed to upscale crop simulations for Denmark. Similarly, [23] found the best agreement of observed and simulated yields with climate and soil data at approximately 1° resolution (approx. 100 km). In the present study, yields were compared to yields of the highest resolution instead of regional statistics. This was done to calculate the direct data aggregation effect, ruling out artefacts in aggregating yield statistics. However, we found ensemble median biases of less than 3% across all resolutions and data type combinations as well as maximum single model biases of less than 4% for up to 25 km resolution and less than 8% for up to 50 km resolution. Depending on the aim, this suggests keeping simulation resolutions roughly in the range of up to 25 or 50 km when the model’s sensitivity to soil data aggregation is not known. By contrast, findings from climate data aggregation studies indicated a reasonable threshold at 100 km resolution for regions in Central Europe [11–12, 30]. Additionally, [25] suggested a resolution of 100 km as reasonable for aggregating soil and climate data for Germany. In general, all of these findings are comparable with the median bias across models of the current work. This emphasizes the dominant influence of spatial soil variability on spatial yield variability and eventually aggregation effects in Europe, being similar to findings by [22]. [28] suggested to adapt the resolution of climate input data to the spatial heterogeneity of relevant variables, e.g. topography. With this approach, they were able to reduce the root mean squared error (RMSE) in winter wheat yield when aggregating climate data. While promising, the performance of the approach remains to be assessed for other input data types and aggregation methods.

#### Patterns in soil and climate aggregation effects

The presented rMAE show large differences across data types and aggregation methods, which are not captured by the regional yield bias when using a model ensemble median. It would thus be intriguing to test, how much single models were influenced by either soil or climate data aggregation. While a detailed assessment of this aspect is beyond the scope of this work, the presented scatter of model aggregation effects, grouped by their magnitude, can serve as an initial indicator. We have shown for all models that the direction and magnitude of the aggregation effect depends on the yield and awc at 1 km resolution, and the change in awc when aggregating soil or soil and climate. This relationship may differ for regions with less precipitation. Still, if yields can be approximated by few variables, mainly soil related variables as in the present study, we hypothesize that the aggregation error can thus be approximated. By knowing the soil properties and their distribution, changes in soil due to aggregation by majority are known. Thus, the direction and magnitude of the aggregation effect may be estimated before conducting large-scale studies. If a model response (e.g. simulated yield) on each soil type is known and the model response to climate is low or climate is spatially homogeneous, then aggregation effects could be estimated by calculating the expected differences from changing spatial soil type composition. However, when extrapolating for different regions, this is restricted by the accuracy and robustness in relating a models yield to the given soil conditions. Other approaches could follow [28], possibly incorporating further or different variables as proxies.

Aggregation effects of soil and climate data were not additive in the present study. However, we showed that combined soil and climate data aggregation can double the aggregation error in parts of the aggregation error distribution. This was due to the different methods of aggregation. Climate data averaging led to a larger proportion of low aggregation errors already at 10 km resolution, while soil aggregation led to a larger fraction of grid cells and years with no error as well as in the fraction of largest errors. Additionally, the maximum increase in the aggregation error due to combined climate and soil data aggregation was specifically observed between the points of inflexion of aggregation errors due to climate aggregation and those due to soil data aggregation. This additional aggregation error could thus possibly serve as an indicator of crop model sensitivity to errors from aggregated soil and climate data.

### Limitations and link to aggregation effect assessment

Firstly, two aggregation methods were applied on two data types. Therefore, climate data averaging effects are not directly comparable to soil data aggregation by majority. For a complete analysis of the variances in aggregation effects, it would thus be necessary to average soil data and select climate time series by majority. However, due to required consistency across variables, soil data averaging was not feasible. Similarly, selecting climate time series by majority is not feasible without previous classification, since all climate time series differ. The presented results therefore primarily demonstrate possible ranges and patterns of aggregation effects. While the aggregation methods used in this work are most common, large-scale studies use a larger range of aggregation and related scaling methods [13–14]. Furthermore, aggregation of other data types than used here may affect the aggregation differently. For instance, a constant management (sowing, fertilization) was used in the present study. However, management may be based on decision rules. Using aggregated management based on these rules at 1 km resolution or applying them directly at coarser resolution (e.g. aggregated climate) could lead to aggregation errors. This remains to be confirmed.

Secondly, crop models were calibrated in order to represent regional long-term mean yields over the entire study area. Thus, models which were tuned to fit observed yields in more detail (e.g. in single years or counties) are possibly less robust to input data aggregation as presented here. Finally, the effects of spatial data aggregation on temporal in- and output patterns are unknown. For instance, temporal scale-invariant time series parameters [77] may be affected by spatial aggregation. As this is potentially relevant for impact assessment, this remains to be investigated.

## Conclusions

We conclude that, when simulating regional water-limited average yields in a temperate humid region, most models are little affected by aggregating soil and/or climate data up to 100 km resolution. However, some models showed considerably larger biases in the range or larger than the inter-annual yield variability. Consequently, models need to be assessed individually for their robustness to climate and soil input data aggregation when simulating regional yields. Furthermore, absolute errors (rMAE) of most models using aggregated soil data are in the range or larger than the inter-annual yield variability or differences between models. Being even larger than aggregation effects from averaged climate data, aggregating soils can thus be a dominant source of uncertainty when assessing spatial yield patterns of heterogeneous regions. Furthermore, simultaneous use of aggregated climate and soil data is likely to increase these aggregation effects even further. However, aggregation effects in yield show distinct patterns depending on the type of data being combined. Large negative aggregation effects were found in areas with soils characterized by high available water holding capacity and large positive aggregation effects in areas with soils of predominantly low available water holding capacity. Considering the regional precipitation pattern, this indicates that the direction and magnitude of aggregation effects may be estimated from a limited number of soil variables.

## Supporting Information

S1 Dataset Minimal dataset with underlying data of presented results. (ZIP)

S1 Fig Supporting Figures A-G. (PDF)

S1 File Equations. (DOCX)

S1 Table Supporting Tables A-D. (PDF)
