# Supplementary material for: Impact of Spatial Soil and Climate Input Data Aggregation on Regional Yield Simulations
Source: PLoS One. 2016 Apr 7;11(4):e0151782. doi: 10.1371/journal.pone.0151782 (PMC4824533; doi:10.1371/journal.pone.0151782)
Supplement: S1 Fig — (PDF) [file pone.0151782.s002.pdf]

Supplementary

Impact of spatial soil and climate input data aggregation on regional yield simulations

Holger Hoffmann*[1], Gang Zhao[1], Senthold Asseng[2], Marco Bindi[3], Christian Biernath[4], Julie Constantin[5], Elsa Coucheney[6], Rene Dechow[7], Luca Doro[8], Henrik Eckersten[9], Thomas Gaiser[1], Balázs Grosz[7], Florian Heinlein[4], Belay T. Kassie[2], Kurt-Christian Kersebaum[10], Christian Klein[4], Matthias Kuhnert[11], Elisabet Lewan[6], Marco Moriondo[12], Claas Nendel[10], Eckart Priesack[4], Helene Raynal[5], P. Paolo Roggero[8], Reimund P. Rötter[13], Stefan Siebert[1], Xenia Specka[10], Fulu Tao[13], Edmar Teixeira[14], Giacomo Trombi[3], Daniel Wallach[5], Lutz Weihermüller[15], Jagadeesh Yeluripati[16], Frank Ewert[1]

[1] Crop Science Group, INRES, University of Bonn, Katzenburgweg 5, 53115 Bonn, DE

[2] Agricultural & Biological Engineering Department, University of Florida, Frazier Rogers Hall, Gainesville, FL 32611, USA

[3] Department of Agri-food Production and Environmental Sciences - University of Florence, Piazzale delle Cascine 18, 50144 Firenze, IT

[4] Institute of Biochemical Plant Pathology, German Research Center for Environmental Health, Ingolstädter Landstraße 1, D 85764 Neuherberg, DE

[5] INRA, UMR 1248 AGIR & UR0875 MIA-T, F-31326 Auzeville, FR

[6] Department of Soil and Environment, Swedish University of Agricultural Sciences, Lennart Hjelms väg 9, 750 07 Uppsala, SE

[7] Thünen-Institute of Climate-Smart-Agriculture, Bundesallee 50, 38116 Braunschweig, DE

[8] Desertification Research Group, Universitá degli Studi di Sassari, Viale Italia 39, 07100 Sassari, IT

[9] Department of Crop Production Ecology, Swedish University of Agricultural Sciences, Ulls väg 16, 750 07 Uppsala, SE

[10] Institute of Landscape Systems Analysis, Leibniz Centre for Agricultural Landscape Research, 15374 Müncheberg, DE

[11] Institute of Biological and Environmental Sciences, School of Biological Sciences, University of Aberdeen, 23 St Machar Drive, Aberdeen AB24 3 UU, Scotland, UK

[12] CNR-Ibimet, Via Caproni 8, 50145, Florence, Italy

[13] Environmental Impacts Group, Natural Resources Institute Finland (Luke), 01370 Vantaa, FI

[14] Systems Modelling Team (Sustainable Production Group), The New Zealand Institute for Plant and Food Research Limited, Canterbury Agriculture & Science Centre, Gerald St, Lincoln 7608, NZ

[15] Agrosphere Institute (IBG-3), Forschungszentrum Jülich GmbH, 52428 Jülich, DE

[16] The James Hutton Institute, Craigiebuckler, Aberdeen AB15 8QH, UK

*Corresponding author:

E-mail: hhoffmann@uni-bonn.de

Figures

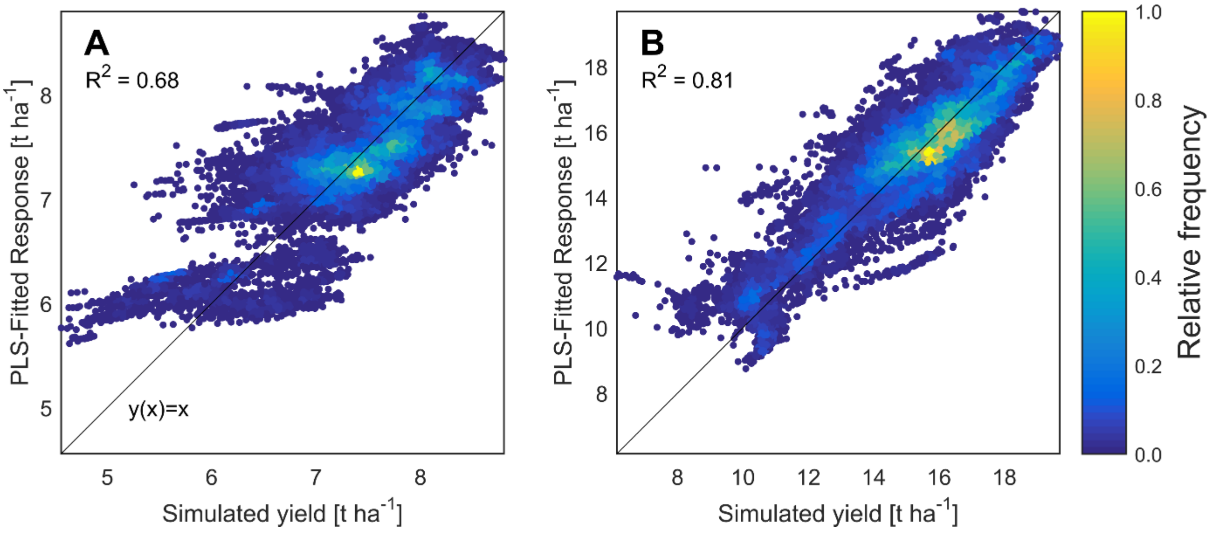

Fig. A. Best fit following PLS-regression of crop yields (average of years) at 1 km soil x 1 km climate resolution (n = 34168) with four explaining variables and four components. (A) Winter wheat (variables: growing season precipitation, available water capacity of soil profile (awc), soil profile depth, topsoil awc). (B) Silage maize (variables: growing season mean daily temperature, awc, soil profile depth, topsoil awc).

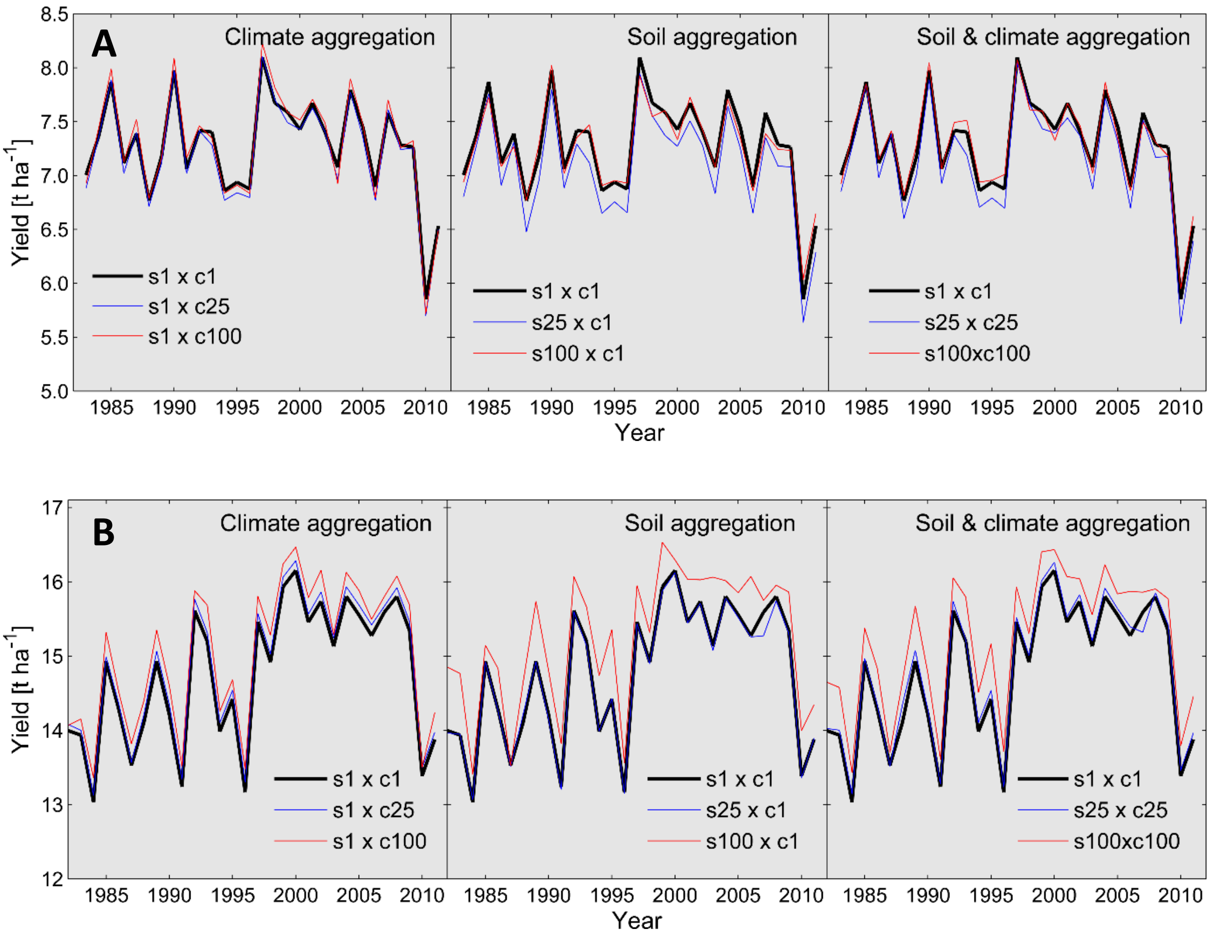

Fig. B. Influence of soil and climate input data aggregation on ensemble and area mean simulated yield. (A) Winter wheat. (B) Silage maize. Legends indicate the resolution of soil (s) and climate (c) input data resolution [km].

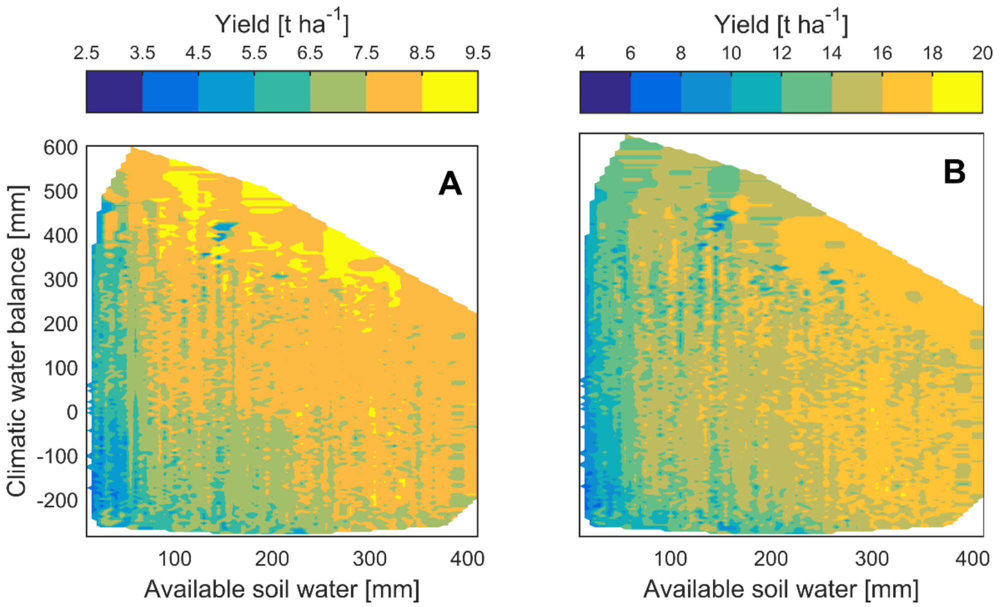

Fig. C. Simulated yield of North Rhine-Westphalia, Germany (1983 - 2011) as related to the plant available water over the profile and climatic water balance (precipitation minus potential evapotranspiration) of the growing season. (A) Winter wheat. (B) Silage maize. The surface was generated from single year yields of 34168 grid cells at 1 km resolution (mean of models).

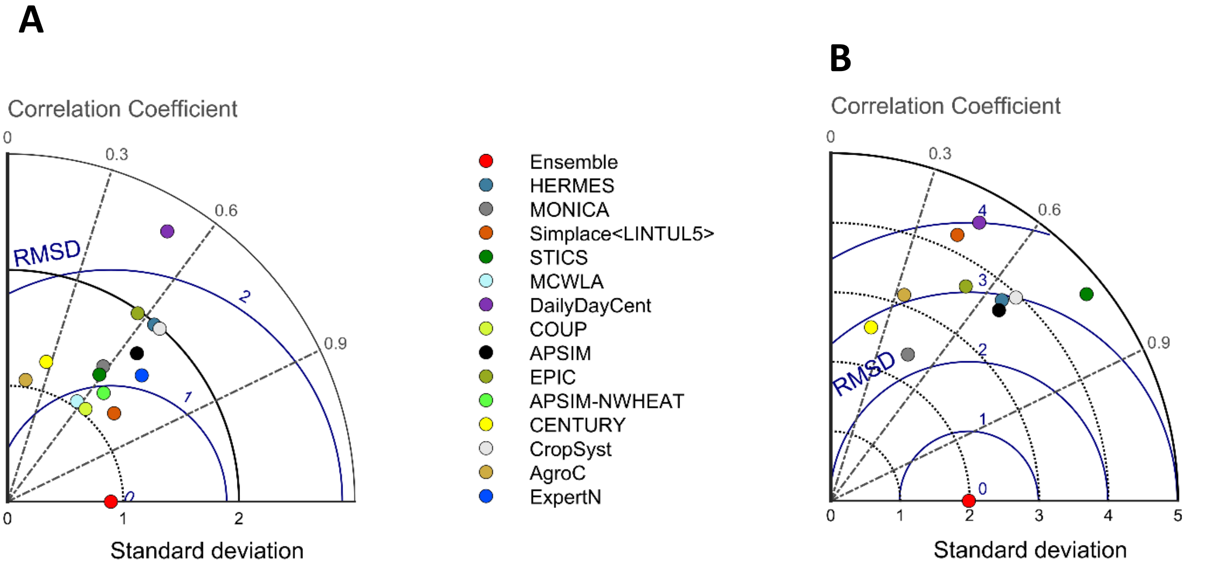

Fig. D. Taylor diagrams [1] of simulated yields from 29 (winter wheat) and 30 (silage maize) years and from 34168 grid cells at 1 km resolution, showing: the standard deviation of each model (σ), the correlation between the models (R) and the centred root mean square difference (RMSD) to the ensemble mean. (A) Winter wheat. (B) Silage maize. Less scatter shows smaller diversity among models and vice versa. RMSD and standard deviation are given in $t\ ha^{-1}$. For each model n = 990,872 and n = 1,025,040 for winter wheat and silage maize, respectively.

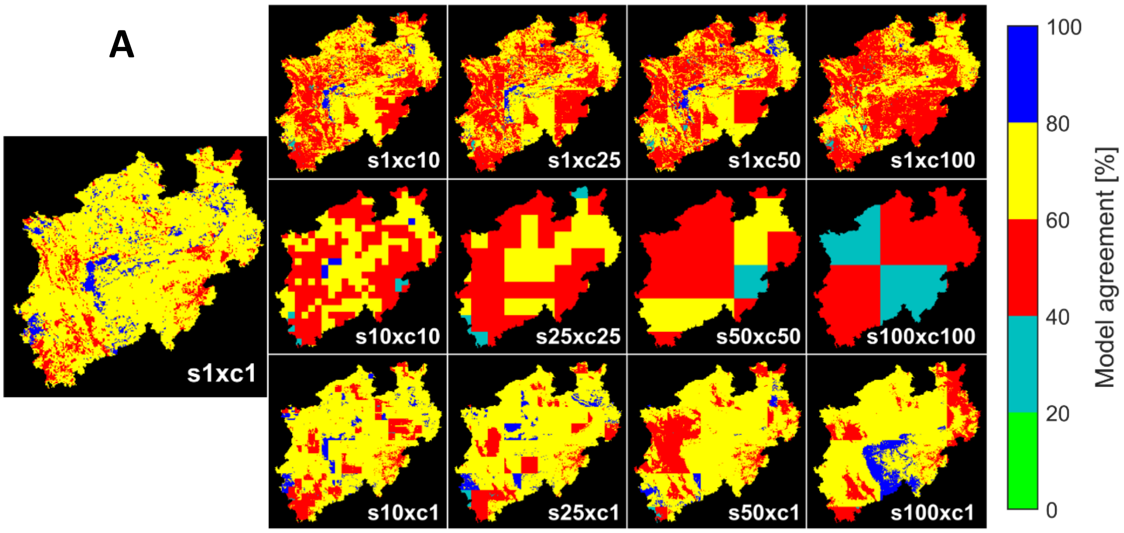

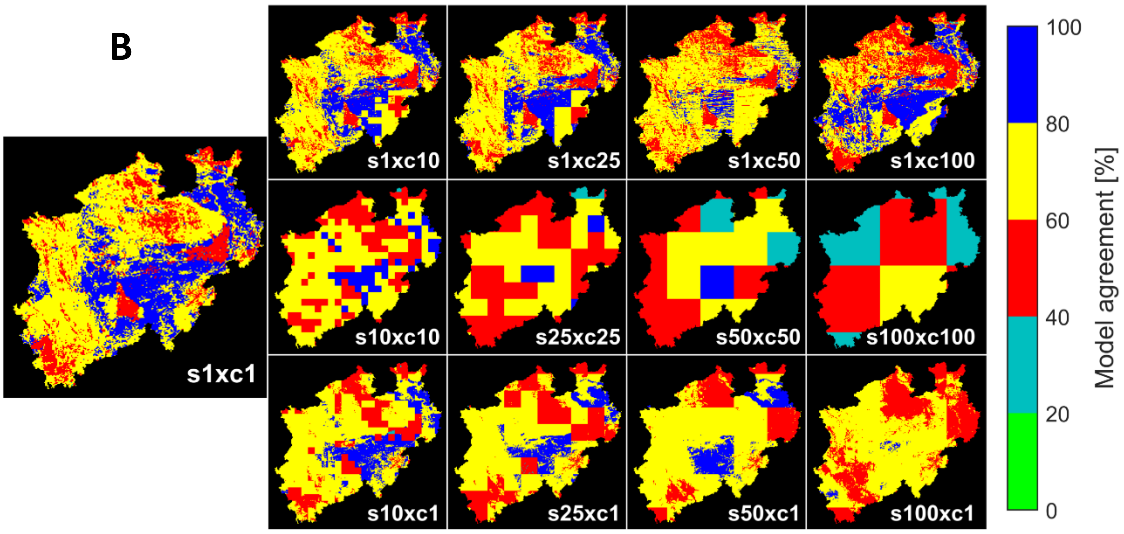

Fig. E. Model agreement in mean simulated yield of North Rhine-Westphalia, Germany (1982 - 2011). (A) Winter wheat. (B) Silage maize.

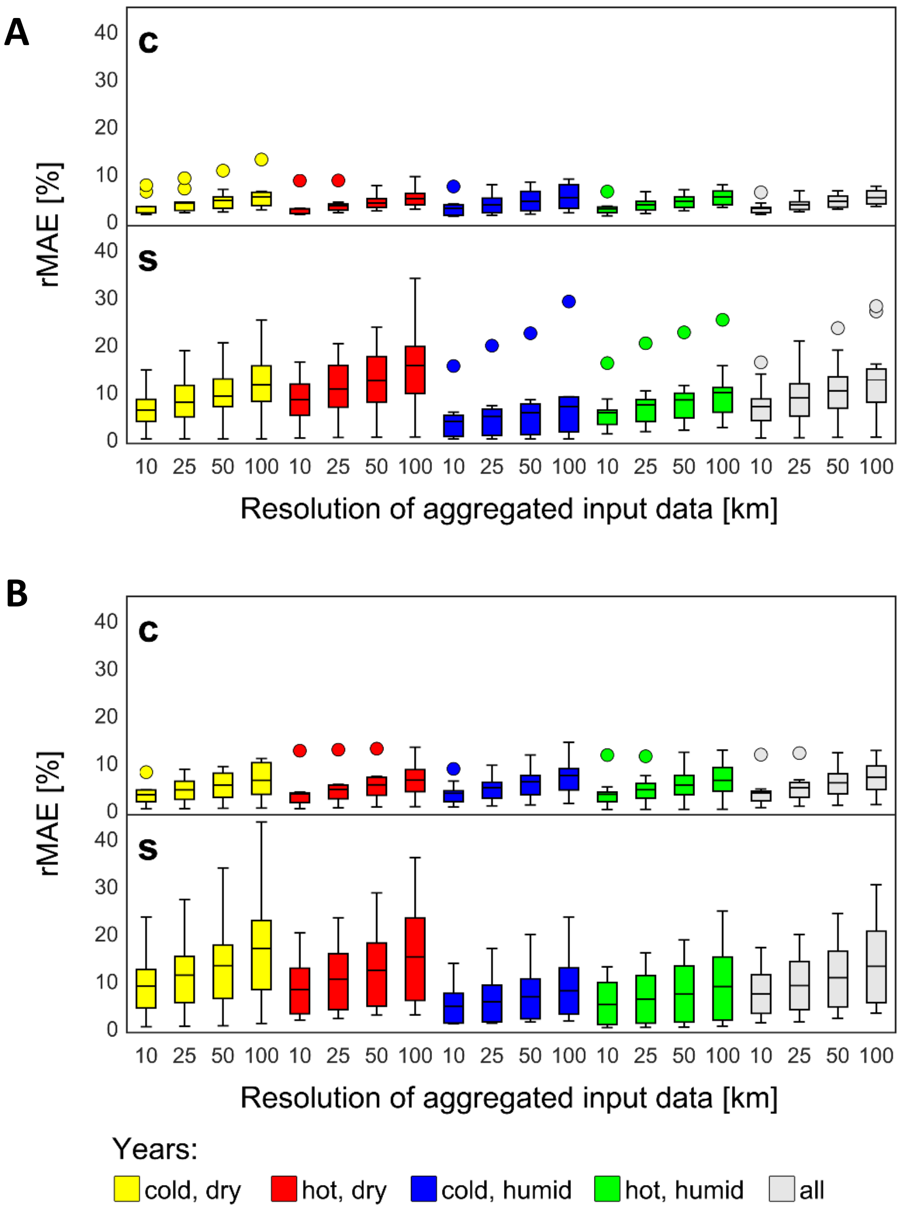

Fig. F. Relative mean absolute error (rMAE) of simulated yield for different spatial resolutions of model input data. (A) Winter wheat. (B) Silage maize. c: aggregated climate x one select soil; s: aggregated soil x average climate time series. The rMAE was calculated from data of extreme years years (see fig. 2) and of all single years. Boxplots show the rMAE calculated from n = 11 single model results (middle line indicates the mean rMAE across models, whiskers are Tukey style and extent to 1.5 times the interquartile range; see [2]).

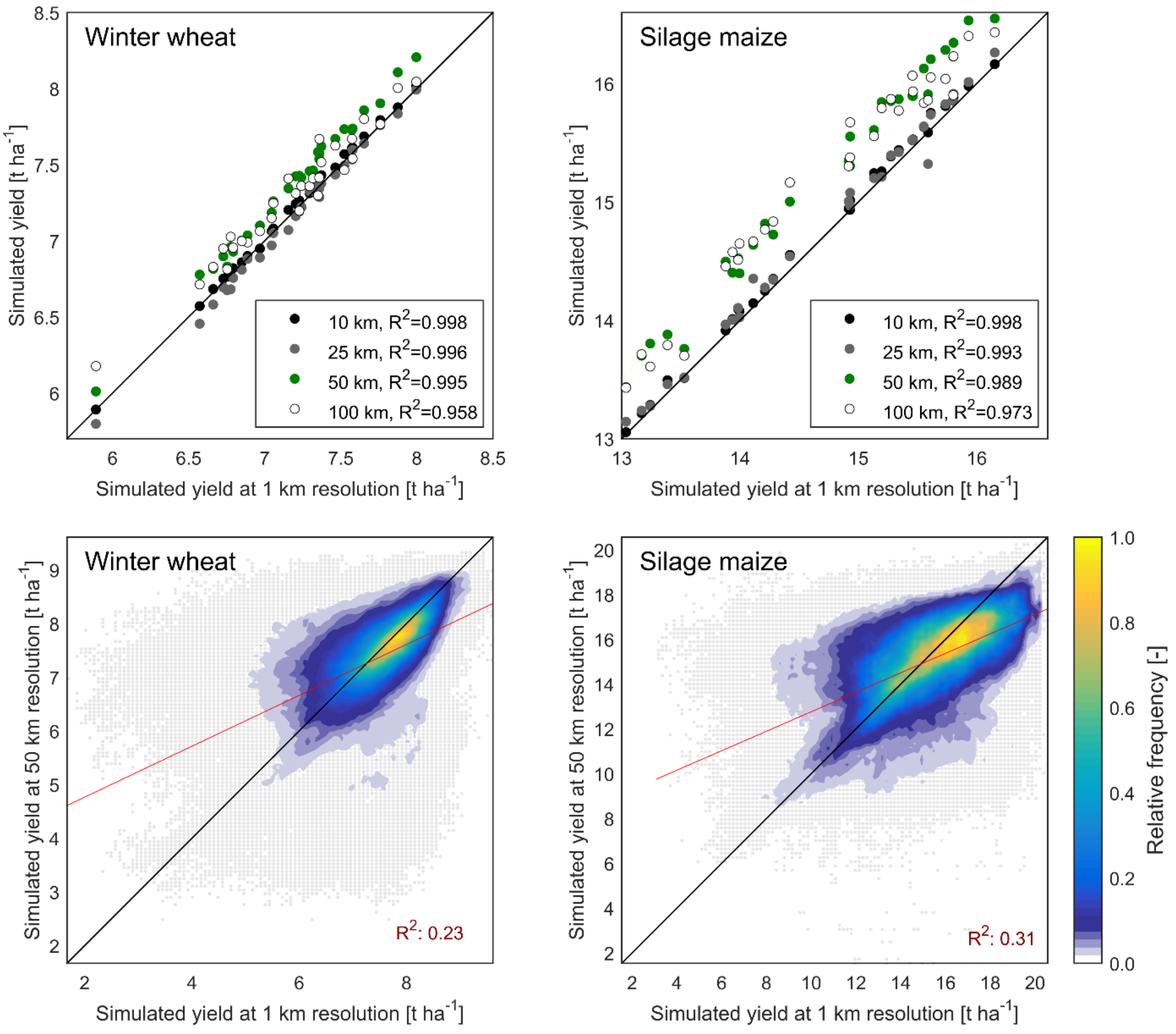

Fig. G. Comparison of simulated winter wheat and silage maize yield from aggregated soil and climate data with simulated yields at 1 km resolution. Upper row: regional mean yields of single years. Bottom row: yields of single cells and single years at 50 km resolution. All values show the model ensemble median. Black line: 1:1-line; red line: linear regression.

References

1. Taylor KE. Summarizing multiple aspects of model performance in a single diagram. J Geophys Res-Atmos. 2001; 106: 7183-7192.
2. Krzywinski M, Altman N. Visualizing samples with box plots. Nature Methods. 2014; 11: 119-120.
