# Supplementary material for: Impact of Spatial Soil and Climate Input Data Aggregation on Regional Yield Simulations
Source: PLoS One. 2016 Apr 7;11(4):e0151782. doi: 10.1371/journal.pone.0151782 (PMC4824533; doi:10.1371/journal.pone.0151782)
Supplement: S1 File — (DOCX) [file pone.0151782.s003.docx]

**Supplementary**

**Impact of spatial soil and climate input data aggregation on regional yield simulations**

Holger Hoffmann*^1^, Gang Zhao^1^, Senthold Asseng^2^, Marco Bindi^3^, Christian Biernath^4^, Julie Constantin^5^, Elsa Coucheney^6^, Rene Dechow^7^, Luca Doro^8^, Henrik Eckersten^9^, Thomas Gaiser^1^, Balázs Grosz^7^, Florian Heinlein^4^, Belay T. Kassie^2^, Kurt-Christian Kersebaum^10^, Christian Klein^4^, Matthias Kuhnert^11^, Elisabet Lewan^6^, Marco Moriondo^12^, Claas Nendel^10^, Eckart Priesack^4^, Helene Raynal^5^, P. Paolo Roggero^8^, Reimund P. Rötter^13^, Stefan Siebert^1^, Xenia Specka^10^, Fulu Tao^13^, Edmar Teixeira^14^, Giacomo Trombi^3^, Daniel Wallach^5^, Lutz Weihermüller^15^, Jagadeesh Yeluripati^16^, Frank Ewert^1^

1 Crop Science Group, INRES, University of Bonn, Katzenburgweg 5, 53115 Bonn, DE

2 Agricultural & Biological Engineering Department, University of Florida, Frazier Rogers Hall, Gainesville, FL 32611, USA

3 Department of Agri-food Production and Environmental Sciences - University of Florence, Piazzale delle Cascine 18, 50144 Firenze, IT

4 Institute of Biochemical Plant Pathology, German Research Center for Environmental Health, Ingolstädter Landstraße 1, D 85764 Neuherberg, DE

5 INRA, UMR 1248 AGIR & UR0875 MIA-T, F-31326 Auzeville, FR

6 Department of Soil and Environment, Swedish University of Agricultural Sciences, Lennart Hjelms väg 9, 750 07 Uppsala, SE

7 Thünen-Institute of Climate-Smart-Agriculture, Bundesallee 50, 38116 Braunschweig, DE

8 Desertification Research Group, Universitá degli Studi di Sassari, Viale Italia 39, 07100 Sassari, IT

9 Department of Crop Production Ecology, Swedish University of Agricultural Sciences, Ulls väg 16, 750 07 Uppsala, SE

10 Institute of Landscape Systems Analysis, Leibniz Centre for Agricultural Landscape Research, 15374 Müncheberg, DE

11 Institute of Biological and Environmental Sciences, School of Biological Sciences, University of Aberdeen, 23 St Machar Drive, Aberdeen AB24 3 UU, Scotland, UK

12 CNR-Ibimet, Via Caproni 8, 50145, Florence, Italy

13 Environmental Impacts Group, Natural Resources Institute Finland (Luke), 01370 Vantaa, FI

14 Systems Modelling Team (Sustainable Production Group), The New Zealand Institute for Plant and Food Research Limited, Canterbury Agriculture & Science Centre, Gerald St, Lincoln 7608, NZ

15 Agrosphere Institute (IBG-3), Forschungszentrum Jülich GmbH, 52428 Jülich, DE

16 The James Hutton Institute, Craigiebuckler, Aberdeen AB15 8QH, UK

*Corresponding author:

E-mail: [hhoffmann@uni-bonn.de](mailto:hhoffmann@uni-bonn.de)

**Equations**

**Soil surface albedo.** Soil surface albedo (α; [-]) was calculated from soil organic content (
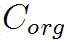
; [g/100 g]) using the following equation (Thomas Gaiser, personal communication):


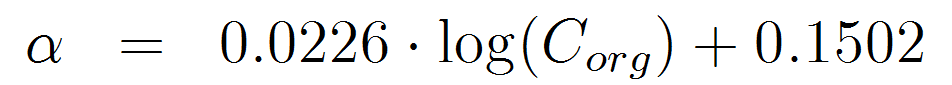
 (A)


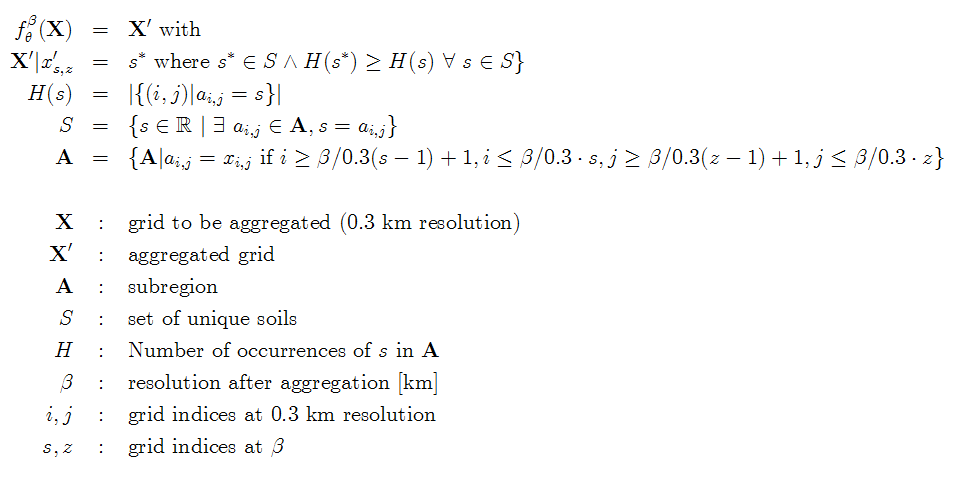
**Grids and aggregation.** We used spatial grids of cells of approximately equal longitudinal and latitudinal side length (<http://spatialreference.org/ref/epsg/31467/>; [1]). Grids of aggregated climate data were obtained by taking the spatial mean of data at 1 km resolution (see [1] for equations). Grids of aggregated soil data were obtained by selecting the dominant soil by area majority. For this purpose, we counted all soil profiles at 0.3 km resolution, which fall into a given cell of a coarser resolution. The most abundant soil (= area majority) was selected to represent the given cell at coarser resolution. For soil aggregation, the following equations were used:

(B)

Five grids of resolutions of 1, 10, 25, 50 and 100 km with the corresponding number of grid cells 34168, 20, 80, 24 and 9 were constructed. Coarser grids were technically set-up starting in the north-west corner of the study region. Empty grid cells (data unavailable) were ignored in the calculations. Maps displaying main regional climate variables are given by [2].

**Variable notation.** A simplified notation will be used in the following, indexing variables in their dimensions
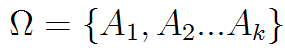
. In the following, equations are conducted over all elements of a given dimension and are applied to all dimensions indicated. For instance, averages are given by


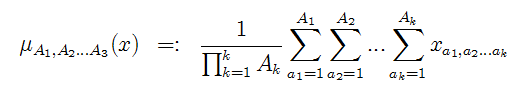
(C)

where the function *µ* averages *x* over dimensions . In the following, these dimensions were considered


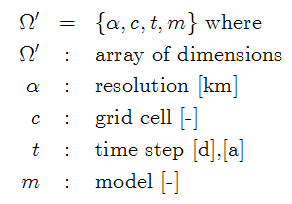
(D)

e.g.
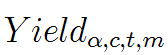
refers to a yield of a given resolution, grid cell, year and model.

**Model agreement.** In order to calculate the spatial agreement of the model ensemble in yields, single model yields were first normalized by the minimum and maximum yield simulated by that model (eq. 5). Subsequently, model agreement Z was obtained by calculating the standard deviation across models and relating it to the maximum possible deviation (eq. 6). Large values of Z indicate better model agreement.


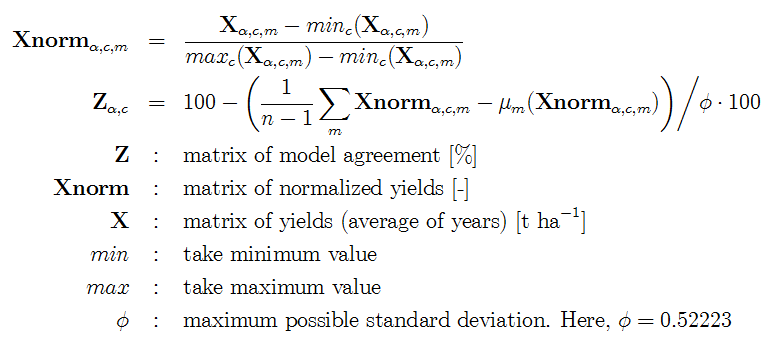
(E)

(F)

**References**

1. Hoffmann H, Zhao G, van Bussel LGJ, Enders A, Specka X, Sosa C, et al. Variability of aggregation effects of climate data on regional yield simulation by crop models. Clim Res. 2015; 65: 53-69.
2. Zhao G, Hoffmann H, Van Bussel LGJ, Enders A, Specka X, Sosa C, et al. Effect of weather data aggregation on regional crop simulation for different crops, production conditions, and response variables. Clim Res. 2015; 65: 141-157.
