# Supplementary material for: Impact of Spatial Soil and Climate Input Data Aggregation on Regional Yield Simulations
Source: PLoS One. 2016 Apr 7;11(4):e0151782. doi: 10.1371/journal.pone.0151782 (PMC4824533; doi:10.1371/journal.pone.0151782)
Supplement: S1 Table — (PDF) [file pone.0151782.s004.pdf]

Supplementary

Impact of spatial soil and climate input data aggregation on regional yield simulations

Holger Hoffmann*[1], Gang Zhao[1], Senthold Asseng[2], Marco Bindi[3], Christian Biernath[4], Julie Constantin[5], Elsa Coucheney[6], Rene Dechow[7], Luca Doro[8], Henrik Eckersten[9], Thomas Gaiser[1], Balázs Grosz[7], Florian Heinlein[4], Belay T. Kassie[2], Kurt-Christian Kersebaum[10], Christian Klein[4], Matthias Kuhnert[11], Elisabet Lewan[6], Marco Moriondo[12], Claas Nendel[10], Eckart Priesack[4], Helene Raynal[5], P. Paolo Roggero[8], Reimund P. Rötter[13], Stefan Siebert[1], Xenia Specka[10], Fulu Tao[13], Edmar Teixeira[14], Giacomo Trombi[3], Daniel Wallach[5], Lutz Weihermüller[15], Jagadeesh Yeluripati[16], Frank Ewert[1]

[1] Crop Science Group, INRES, University of Bonn, Katzenburgweg 5, 53115 Bonn, DE

[2] Agricultural & Biological Engineering Department, University of Florida, Frazier Rogers Hall, Gainesville, FL 32611, USA

[3] Department of Agri-food Production and Environmental Sciences - University of Florence, Piazzale delle Cascine 18, 50144 Firenze, IT

[4] Institute of Biochemical Plant Pathology, German Research Center for Environmental Health, Ingolstädter Landstraße 1, D 85764 Neuherberg, DE

[5] INRA, UMR 1248 AGIR & UR0875 MIA-T, F-31326 Auzeville, FR

[6] Department of Soil and Environment, Swedish University of Agricultural Sciences, Lennart Hjelms väg 9, 750 07 Uppsala, SE

[7] Thünen-Institute of Climate-Smart-Agriculture, Bundesallee 50, 38116 Braunschweig, DE

[8] Desertification Research Group, Universitá degli Studi di Sassari, Viale Italia 39, 07100 Sassari, IT

[9] Department of Crop Production Ecology, Swedish University of Agricultural Sciences, Ulls väg 16, 750 07 Uppsala, SE

[10] Institute of Landscape Systems Analysis, Leibniz Centre for Agricultural Landscape Research, 15374 Müncheberg, DE

[11] Institute of Biological and Environmental Sciences, School of Biological Sciences, University of Aberdeen, 23 St Machar Drive, Aberdeen AB24 3 UU, Scotland, UK

[12] CNR-Ibimet, Via Caproni 8, 50145, Florence, Italy

[13] Environmental Impacts Group, Natural Resources Institute Finland (Luke), 01370 Vantaa, FI

[14] Systems Modelling Team (Sustainable Production Group), The New Zealand Institute for Plant and Food Research Limited, Canterbury Agriculture & Science Centre, Gerald St, Lincoln 7608, NZ

[15] Agrosphere Institute (IBG-3), Forschungszentrum Jülich GmbH, 52428 Jülich, DE

[16] The James Hutton Institute, Craigiebuckler, Aberdeen AB15 8QH, UK

*Corresponding author:

E-mail: hhoffmann@uni-bonn.de

Tables

Table A. Aggregated model input data. If not specified, soil data was available for each soil layer.

Domain	Variable	Unit
Climate	Precipitation, daily sum [a]	mm d^{-1}
Climate	Air temperature at 2 m above ground, daily minimum, mean, maximum [a]	°C
Climate	Global radiation, daily sum [a]	MJ m^{-2} d^{-1}
Climate	Wind speed, daily average [a]	m s^{-1}
Climate	Relative humidity, daily average	%
Soil	Soil type [b,c]	-
Soil	Number of layers[c]	-
Soil	Layer depth (D) and thickness (T)	m
Soil	Water capacity at air dryness (assumed to be 50 % of wilting point) [d]	m^3 m^{-3}
Soil	Water capacity at wilting point (WCWP, pF 4.2) [d]	m^3 m^{-3}
Soil	Water capacity at field capacity (WCFC, pF 2.5)[d]	m^3 m^{-3}
Soil	Water capacity at saturation (WCST, pF 0) [d]	m^3 m^{-3}
Soil	Air Capacity [d,e]	m^3 m^{-3}
Soil	Clay fraction of fine earth	%
Soil	Silt fraction of fine earth	%
Soil	Sand fraction of fine earth	%
Soil	Gravel content (mass)	%
Soil	Gravel content (volumetric) [f]	%
Soil	Bulk density of fine earth (excluding gravel)	g cm^{-3}
Soil	Bulk density of total soil (including gravel) [g]	g cm^{-3}
Soil	Organic carbon content [h]	[%]

Soil	C:N-ratio [h,i]	-
Soil	pH	-
Soil	surface albedo [c,j]	-
Soil	Calcium Carbonate content	%
Soil	Plant available water capacity (awc) [k]	mm

a) See [1] for a more detailed description of data origin and processing. b) This is metadata. c) One value per profile. d) Original values from fine earth fraction (Clay + Silt + Sand = 100 %) were corrected for gravel content. e) calculated as difference WCST – WCFC. f) Calculated from gravel content on mass/mass ratio via Nomogram [2]. g) Approximated via Nomogramm (Poesen & Lavee, 1994). h) For deeper layers, approximated via pedotransfer functions [6-7]. i) Top soil layer C:N-ratio was set to 10. j) Estimated from soil organic carbon of soil top layer (R^2=0.97), eq. 1. k) awc = (WCFC-WCWP)·T·10.

Table B. Crop model settings and assumptions.

Domain	Unit	Winter Wheat	Silage Maize
Sowing date	DOY[a]	274	110
Harvest date (calibration)	DOY[a]	213	263
Harvest date (simulation)	DOY[a]	at simulated maturity	at simulated maturity
Average Yield[b] (calibration)	t ha^{-1}	7.2	14.3
Max. rooting depth	m	1.5	1.5
Time of ploughing	-	autumn	autumn
Planting density	m^{-2}	400	10
Sowing depth	m	0.04	0.06
Initial soil moisture relative to available field capacity[c]	%	50	80
Initial Nmin[d]	kg ha^{-1}	56	56
Nitrogen fertilization	kg ha^{-1}	130, 52, 26	30, 208
Date of fertilization	DOY[a]	60, 105, 152	91, 152

[a] Day of the year of a non-leap year. [b] Area weighted average yield derived from county statistics, moisture content: 0 %. [c] Set for each soil layer. [d] Total mineral Nitrogen of the soil profile. Values differ with soil layer.

Table C. Effect of aggregation on soil (available water capacity) and climate data (annual precipitation and mean temperature) spatial statistics. Values were calculated across the space and for climate data subsequently averaged over the years.

Data type	Variable	Resolution	Min	Median	Max	standard deviation	Skewness [-]	Kurtosis [-]
Soil	AWC[a]	0.3	10	160	412	78	0.23	2.63
Soil	AWC[a]	1	10	160	412	83	0.32	2.64
Soil	AWC[a]	10	16	160	412	89	0.36	2.52
Soil	AWC[a]	25	22	171	347	91	0.25	2.13
Soil	AWC[a]	50	22	178	347	81	0.32	2.61
Soil	AWC[a]	100	22	182	347	100	0.46	2.24
Climate	T2[b]	1	5.6	10.0	11.6	1.0	-0.96	3.44
Climate	T2[b]	10	6.7	9.9	11.3	0.9	-0.89	3.14
Climate	T2[b]	25	7.2	9.9	10.9	0.9	-0.85	2.93
Climate	T2[b]	50	7.6	9.8	10.6	0.8	-0.92	2.98
Climate	T2[b]	100	8.4	9.8	10.4	0.7	-0.64	1.85
Climate	An. Pr.[c]	1	548	846	1592	183	0.99	3.39
Climate	An. Pr.[c]	10	587	834	1408	177	0.97	3.24
Climate	An. Pr.[c]	25	630	831	1329	163	0.96	3.19
Climate	An. Pr.[c]	50	669	833	1185	139	1.02	3.05
Climate	An. Pr.[c]	100	703	823	1081	113	1.26	2.99

[a] Available water capacity corrected for gravel content [mm]; [b] Daily mean air temperature at 2 m aboveground [°C]; [c] Annual precipitation [mm]

Table D. Characterization of differences in yield due to data aggregation (ΔY) as related to yield (Y), soil water holding capacity (SWHC), climatic water balance during the growing season (CWB) and the corresponding differences from coarser resolutions to 1 km resolution due to data aggregation (ΔY, ΔSWHC, ΔCWB), shown as parallel coordinates plot. The ΔY of each model, crop and aggregation type (soil: aggregation of soil at 1 km climate resolution; climate: aggregation of climate at 1 km soil resolution; soil x climate: simultaneous aggregation of soil and climate) was grouped as follows. LL: $\Delta Y<\mu-2\sigma$; L0: $\mu-2\sigma<\Delta Y<\mu-\sigma$; MM: $\mu-\sigma<\Delta Y<\mu+\sigma$; H0: $\mu+\sigma<\Delta Y<\mu+2\sigma$; HH: $\Delta Y>\mu+2\sigma$ where μ: mean of ΔY and σ: standard deviation of ΔY. W: winter wheat; M: silage maize; s: soil aggregation at 1 km climate; c: climate aggregation at 1 km soil; sxc: aggregation of soil x climate; n: number of cells and years in group. Values were taken from resolutions 10, 25, 50 and 100 km.

			**Average**							**Standard deviation**					
**Crop**	**type**	**group**	***n***	***Y***	***ΔY***	***SWHC***	***ΔSWHC***	***CWB***	***ΔCWB***	***Ys***	***ΔY***	***SWHCs***	***ΔSWHC***	***CWBs***	***ΔCWB***
W	s	LL	145555	8.1	**-3.8**	190	-100	-63	0	41590	0.7	1.9	30	25	22
W	s	L0	232578	7.7	**-2**	180	-45	-63	0	112470	0.6	1.1	18	39	15
W	s	MM	3210481	7.4	**-0.1**	161	8	-27	0	221270	0.5	0.4	4	2	5
W	s	H0	227006	5.9	**1.8**	133	68	-59	0	85660	0.9	1.3	20	25	19
W	s	HH	145147	4.3	**3.7**	114	93	-63	0	47770	1.2	2	43	36	26
W	c	LL	105813	7.8	**-2.1**	113	0	-11	-38	38550	0.8	0.9	29	0	32

W	c	L0	267803	7.5	**-1.1**	127	0	-21	-23	73350	0.7	0.7	21	0	23
W	c	MM	3122257	7.3	**0**	165	0	-34	-6	382260	0.5	0.3	4	0	5
W	c	H0	265441	6.4	**1**	147	0	-34	1	92300	0.6	0.5	18	0	22
W	c	HH	114234	5.3	**2.3**	144	0	-43	6	31210	1	1.1	28	0	43
W	sxc	LL	144424	8.1	**-3.9**	175	-86	-49	-12	34830	0.6	1.8	33	39	36
W	sxc	L0	282341	7.8	**-2.1**	166	-36	-45	-10	74280	0.6	1	21	30	23
W	sxc	MM	3110315	7.5	**0**	162	8	-29	-7	128570	0.5	0.2	4	2	6
W	sxc	H0	282780	5.7	**2.1**	135	57	-51	-4	62230	0.8	1.1	19	28	22
W	sxc	HH	141696	4.1	**3.9**	113	85	-54	-5	36830	1.2	1.8	37	37	36
M	s	LL	107675	17.3	**-8.2**	190	-91	-76	0	56570	2.6	3.3	31	42	35
M	s	L0	187567	16.2	**-4**	189	-55	-73	0	122470	2.7	1.9	18	38	17
M	s	MM	2700401	15	**0.1**	159	7	-27	0	1351520	2.5	0.2	4	2	4
M	s	H0	207316	12.2	**4.4**	138	73	-74	0	158010	3	2.3	23	33	21
M	s	HH	126193	8.7	**8.7**	113	93	-73	0	64450	3.6	3.4	44	45	38
M	c	LL	74157	15.6	**-4.5**	134	0	-25	-25	49350	2.7	2.2	28	0	32
M	c	L0	222180	15.3	**-1.9**	142	0	-26	-17	139780	2.6	0.7	22	0	16

M	c	MM	2686404	15	**0.1**	163	0	-36	-7	1344610	2.5	0.2	4	0	6
M	c	H0	233368	12.7	**2.3**	142	0	-31	-7	140380	2.5	0.8	28	0	40
M	c	HH	114427	10.4	**5**	147	0	-39	-1	61220	3.4	2.7	37	0	49
M	sxc	LL	98476	17.4	**-8.5**	184	-86	-67	-7	53070	2.6	3.1	25	41	32
M	sxc	L0	228541	16.3	**-4.2**	180	-44	-55	-5	122800	2.6	1.8	18	34	15
M	sxc	MM	2629461	15.1	**0.1**	161	7	-29	-7	1305030	2.5	0.2	4	2	4
M	sxc	H0	241814	11.6	**4.8**	135	59	-55	-9	130600	2.7	1.8	24	28	32
M	sxc	HH	131946	8.1	**9.2**	114	83	-62	-9	68920	3.3	3.1	41	46	31

References

1. Zhao G, Siebert S, Enders A, Rezaei EE, Yan C, Ewert F. Demand for multi-scale weather data for regional crop modelling. Agr For Meteorol. 2015; 200: 156-171.
2. Torri D, Poesen J, Monaci F, Busoni E. Rock fragment content and fine soil bulk density. Catena. 1994; 23: 65-71.
